# A HIF-1 network reveals characteristics of epithelial-mesenchymal transition in acute promyelocytic leukemia

**DOI:** 10.1186/s13073-014-0084-4

**Published:** 2014-12-01

**Authors:** Stefano Percio, Nadia Coltella, Sara Grisanti, Rosa Bernardi, Linda Pattini

**Affiliations:** Department of Electronics, Information and Bioengineering, Politecnico di Milano, piazza Leonardo da Vinci 32, 20133 Milan, Italy; Division of Molecular Oncology, San Raffaele Scientific Institute, Via Olgettina 60, 20132 Milan, Italy; Leukemia Unit, IRCCS Ospedale San Raffaele, Via Olgettina 60, 20132 Milan, Italy

## Abstract

**Background:**

Acute promyelocytic leukemia (APL) is a sub-type of acute myeloid leukemia (AML) characterized by a block of myeloid differentiation at the promyelocytic stage and the predominant t(15:17) chromosomal translocation. We have previously determined that cells from APL patients show increased expression of genes regulated by hypoxia-inducible transcription factors (HIFs) compared to normal promyelocytes. HIFs regulate crucial aspects of solid tumor progression and are currently being implicated in leukemogenesis.

**Methods:**

To investigate the contribution of hypoxia-related signaling in APL compared to other AML sub-types, we reverse engineered a transcriptional network from gene expression profiles of AML patients’ samples, starting from a list of direct target genes of HIF-1. A HIF-1-dependent subnetwork of genes specifically dysregulated in APL was derived from the comparison between APL and other AMLs.

**Results:**

Interestingly, this subnetwork shows a unique involvement of genes related to extracellular matrix interaction and cell migration, with decreased expression of genes involved in cell adhesion and increased expression of genes implicated in motility and invasion, thus unveiling the presence of characteristics of epithelial-mesenchymal transition (EMT). We observed that the genes of this subnetwork, whose dysregulation shows a peculiar pattern across different AML sub-types, distinguish malignant from normal promyelocytes, thus ruling out dependence on a myeloid developmental stage. Also, expression of these genes is reversed upon treatment of APL-derived NB4 cells with all-*trans* retinoic acid and cell differentiation.

**Conclusions:**

Our data suggest that pathways related to EMT-like processes can be implicated also in hematological malignancies besides solid tumors, and can identify specific AML sub-types.

**Electronic supplementary material:**

The online version of this article (doi:10.1186/s13073-014-0084-4) contains supplementary material, which is available to authorized users.

## Background

Acute promyelocytic leukemia (APL) is the M3 sub-type of acute myeloid leukemia (AML) according to the French American British (FAB) classification. AMLs are a heterogeneous class of hematologic malignancies characterized by a block of differentiation at different stages of myeloid lineage specification and abnormal proliferation and self-renewal of hematopoietic cells in the bone marrow and blood [1].

APL is characterized by a differentiation arrest at the promyelocytic stage and by the specific t(15;17) chromosomal translocation, which fuses the promyelocytic leukemia gene (*PML*) and the retinoic acid receptor alpha gene (*RARA*), which together encode the oncogenic PML-RARA fusion protein [2].

The hypoxia-inducible transcription factor HIF-1 is a master regulator of cell responses to hypoxia and its pathways are often up-regulated in solid tumors, where it is associated with metastasis and poor prognosis [3]. In solid tumors, HIF-1 activates a wide range of adaptive responses like anaerobic metabolism, migration, invasion, metastasis and angiogenesis [4,5], while its involvement in leukemogenesis is less studied. It was recently shown that the HIF-1α gene is expressed in the stem cell compartment of mouse lymphoma and human AML [6], and that it plays a crucial role in promoting the maintenance of leukemia stem cells in AML and chronic myeloid leukemia [6,7]. The involvement of the HIF-1 network in leukemogenesis remains, however, to be fully characterized along with its specific contribution to distinct leukemia subtypes.

We have previously found that, in the M3 subtype of AML, HIF-1α is activated by the fusion protein PML-RARα throughout the leukemia bulk, where it regulates not only self-renewal of leukemia stem cells but also cell migration, chemotaxis and neo-angiogenesis [8].

Gene expression profiling can improve our comprehension of altered transcriptional regulation in disease, thus allowing the identification of genes related to pathophysiological mechanisms, while at the same time addressing the inherent heterogeneity of oncologic pathologies. To further elucidate the specificity of the hypoxia response in APL with respect to other AML subtypes, we constructed a relevance network [9] from gene expression profiles of two independent publicly available data sets of AML samples, using a list of HIF-1-direct targets as a seed. To increase specificity, only interactions confirmed in both data sets have been retained. Further selection of the genes differentially expressed in APL samples versus other AML subtypes allowed us to delineate a key HIF-1-dependent gene module in AML, which shows a specific dysregulation that typifies APL. Interestingly, this APL-specific gene signature, configured as a network module, encompasses genes mainly involved in extracellular matrix (ECM) interaction, with decreased expression of genes involved in cell adhesion and increased expression of genes implicated in motility and invasion; these signalling effectors with their specific and coordinated dysregulation pinpoint an epithelial-mesenchymal transition (EMT)-like gene expression program.

## Methods

### Acute myeloid leukemia gene expression data sets

We analyzed gene expression profiles belonging to two independent clinical data sets of AML samples publicly available. The first data set was retrieved from the Gene Expression Omnibus (GEO) repository (accession number GSE1159). It contains transcriptomic profiles of bone marrow or peripheral blood from 293 subjects with different FAB classifications (19 M3 samples) hybridized to the Affymetrix Human Genome U133A Array. The second data set was retrieved from The Cancer Genome Atlas (TCGA) Data Portal (accession LAML). It contains samples of bone marrow from 197 patients (20 M3 samples) analyzed using the Affymetrix Human Genome U133 Plus 2.0 Array; for 178 of these samples (16 M3 samples) transcriptome deep sequencing (RNA-Seq) data were also available, obtained with the Illumina GAllx platform.

### Data preprocessing

Since the data sets were analyzed through different platforms, only the 22,215 probe sets common to the two microarray types were considered in our analysis. The level of expression of each probe set was centered on its mean intensity computed across all samples and logarithmically (base 2) transformed. Variability across the samples was assessed by means of the MATLAB (The MathWorks, Inc., Natick, MA, USA) function *geneentropyfilter*, removing probe sets with entropy values less than the 10th percentile. Transcripts that were neither informative (due to the low variability) nor differentially expressed in the comparison between APL and the other AML subtypes were discarded. For RNA-Seq data, RPKM (reads per kilobase per million mapped reads) values at the gene level were used.

### Network analysis

For both data sets, adjacency matrices containing the mutual information for pairwise gene dependencies were computed by means of ARACNe, a specific tool within the GeWorkbench v2.4.1 framework [10], providing the list of the 119 *bona fide* HIF-1 targets (Additional file 1) as a starting point and selecting the adaptive partitioning algorithm.

A consensus network was then obtained selecting only interactions that were significant in both the adjacency matrices (the significance thresholds were set to *P*-value <10e-15 in the first data set and *P*-value <10e-10 in the second data set to take into account the different numbers of samples).

The overall AML network was further pruned leaving only APL dysregulated transcripts (differentially expressed with FDR adjusted *P*-value <0.05 in the *t*-tests accomplished for both the data sets) to obtain the APL specific subnetwork.

The gene networks were visualized by means of the software Cytoscape v2.3 [11].

### Comparison between normal and leukemic promyelocytes

Gene expression data were obtained for five normal promyelocytes (PM) and 14 APL samples from the GEO data series GSE12662 acquired using the Affymetrix Human Genome U133 Plus 2.0 Array platform. The same probe sets found for the APL-specific gene signature were analyzed for this comparison, accomplished by means of unsupervised hierarchical clustering (correlation coefficient metric and average linkage) and principal component analysis in the MATLAB environment.

### MicroRNA analysis

MicroRNA expression data were available for the TCGA data set. The Pearson correlation coefficient was computed between transcripts found down-regulated in the APL subnetwork and microRNAs up-regulated (*t*-test, Bonferroni corrected *P*-value <0.05) in APL versus other AML subtypes. The predictor MiRanda v.3.3a [12] was exploited to assess the proportion of microRNA-target relationships with a recognizable binding site.

### Annotation analysis

The annotation enrichment analysis was performed using David (The Database for Annotation, Visualization and Integrated Discovery) [13,14].

### Patients’ samples

Bone marrow samples from AML patients containing primary leukemia blasts were collected upon written informed consent in accordance with the Declaration of Helsinki by the Hematology and Bone Marrow Transplantation Unit at IRCCS Ospedale San Raffaele and stored at OSR AML Bio Bank. The study was approved by the Institutional Review Boards of San Raffaele Scientific Institute, Milan.

Four bone marrow samples containing >70% leukemic blasts were selected for each leukemia subtype analyzed (M3 and M5, according to the FAB classification).

### Cell culture and reagents

NB4 APL cells were maintained in RPMI 1640 medium supplemented with 10% fetal bovine serum and antibiotics (Lonza Group Ltd, Basel, Switzerland). HEK-293 T embryonic kidney cells were maintained in IMDM medium supplemented with 10% fetal bovine serum and antibiotics. All cell lines were maintained at 37°C in humidified atmosphere containing 5% CO_2_. Experiments under hypoxic conditions (0.2% O_2_) were performed in a hypoxia workstation (Invivo2 400, Ruskinn Technology, Ltd., Bridgend, South Wales, UK).

The RNA antagonists EZN-2968 (a locked nucleic acid-modified oligonucleotide, LNA-ON, for HIF-1α) and EZN-3088 (control LNA-ON for HIF-1α) [15] were provided by Belrose Pharma Inc., Princeton, NJ, USA, and used in accordance with the manufacturer’s instructions. EZN-2968 and EZN-3088 were transfected in an Amaxa™ 4D-Nucleofector™ System (Lonza).

### Lentiviral vectors

GIPZ HIF-1α small hairpin RNA (shRNA) or control shRNA plasmids were from Open Biosystems, GE Healthcare Dharmacone, Lafayette, CO, USA. Lentiviral vectors were obtained by HEK-293 T transfection with calcium phosphate and subsequent concentration as previously described [16]. NB4 cells were transduced with concentrated vectors by spinoculation, and sorted for GFP expression at least 2 weeks post-infection.

### Real-time PCR

RNA was isolated with the RNeasy mini kit (Qiagen Inc., Valencia, CA, USA) from cell lines and with the ReliaPrep™ RNA Cell Miniprep System (Promega Corporation, Madison, WI, USA) from bone marrow samples of AML patients. cDNA was obtained by retro-transcription of 1 to 2 μg total RNA using Advantage RT for PCR Kit (Takara Bio Europe/Clontech, Saint-Germain-en-Laye, France) or SuperScript® III First-Strand Synthesis SuperMix (Invitrogen, Thermo Fisher Scientific Inc., Waltham, MA, USA) and analyzed by real-time PCR with TaqMan assay using a 7900 Fast-Real Time PCR System (Applied Biosystems, Thermo Fisher Scientific Inc., Waltham, MA, USA).

All probes for TaqMan assays were purchased from Applied Biosystems. 18S was used as an internal control. The relative expression of different cDNAs was calculated using the 2^-ΔΔCt^ method except for assessing the relative expression of *MMP2*, *KRT18*, *IGFBP2*, *ITGB2*, *HMOX1*, *LRP1* and *TWIST1* in AML samples, which was calculated by the 2^-ΔCt^ method.

## Results and discussion

### HIF-1 downstream network inference in acute myeloid leukemia

With the aim of elucidating the contribution of HIF-1 signaling in APL, we used a list of direct target genes of HIF-1 characterized by hypoxia-dependent transcriptional induction and the presence of functional hypoxia responsive elements in their promoters validated by HIF-1 chromatin immunoprecipitation to examine the transcriptional networks linked to hypoxia signaling. Using these 119 genes as a seed (Additional file 1), we reconstructed two distinct transcriptional networks based on two data sets of gene expression profiles in AML: the first data set includes 293 AML samples, out of which 19 are classified as M3, analyzed by Affymetrix HG-U133A Array [17]; the second data set contains 197 AML samples, out of which 20 are classified as M3, analyzed by Affymetrix HG-U133 Plus 2.0 Array (TCGA - Acute Myeloid Leukemia). Only probe sets present in both platforms were processed. Moreover, probe sets showing low variability across the samples, assessed through an entropy measurement [18], and probe sets that were not significant in the comparison between APL samples and other AML subtypes were discarded.

Network identification was accomplished through ARACNe [19], a reverse engineering method of regulatory network reconstruction that exploits the mutual information (MI) operator to estimate the pairwise correlation between transcripts. The significance threshold for MI was set to *P* <10e-15 for the first data set and *P* <10e-10 for the second data set, in order to obtain a comparable number of nodes in the two parallel analyses (3,865 and 3,768, respectively). Then, to increase the reliability of the results, we retained only pairwise interactions occurring in both networks.

**Figure 1:**
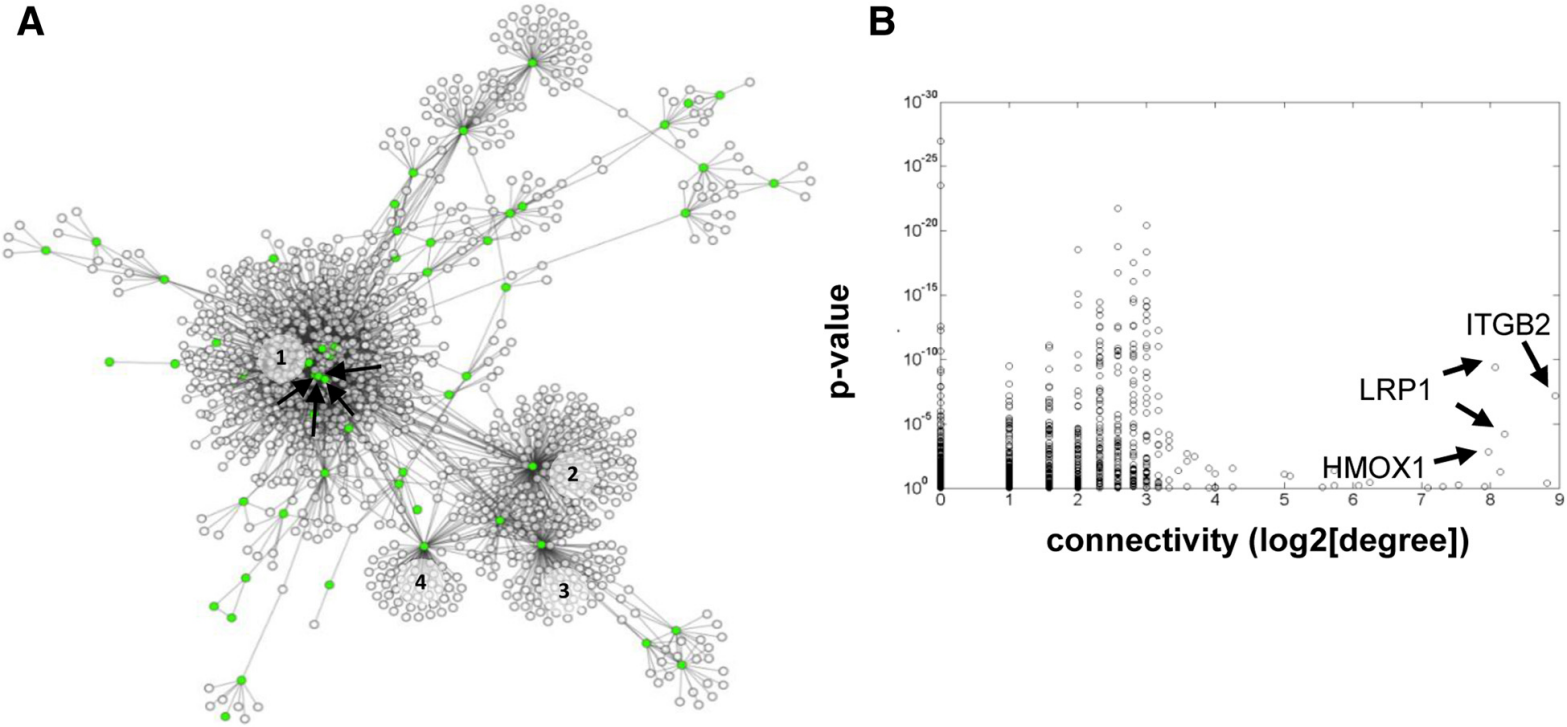
**Reconstructed AML network starting from HIF-1 direct target genes. (A)** Transcriptional network of gene interactions corresponding to pairwise dependencies that were found statistically significant in both gene expression data sets of AML samples. Each circle represents a transcript (probe set). Transcripts corresponding to known HIF-1 direct target genes are highlighted in green. The largest module (1) is enriched for the Gene Ontology terms 'defense response', 'actin cytoskeleton', and 'integral to plasma membrane'; module 2 is enriched for 'ribonucleoprotein complex', 'chaperone', and 'mitochondrion'; module 3 is enriched for 'translational elongation' and 'ribosome'; and module 4 is enriched for 'ncRNA metabolic process'*.* **(B)** Significance in differential expression versus connectivity. For each node of the network, the *P*-value of the *t*-test for the comparison of APL with other AML subtypes is reported against its degree (number of nodes directly connected). *ITGB2*, *LRP1* (two transcripts) and *HMOX1*, indicated by arrows in both panels, emerge as hubs of the network and are highly dysregulated as well.

The consensus network that was obtained with this analysis is shown in Figure 1A: it contains 3,405 edges for 1,908 nodes, out of which 94 are probe sets corresponding to 52 HIF-1 direct targets (highlighted in green).

Given the modularity of the network, we analyzed the annotations for the most crowded modules. The main moule is significantly enriched for the Gene Ontology terms 'defense response' (*P* =1.84e-16), 'actin cytoskeleton' (*P* =1.14e-05), and 'integral to plasma membrane' (*P* =1.67e-05). Two numerous modules communities are centered on two distinct transcripts of the nucleophosmin 1 gene (*NPM1*), which is associated with AML development [20], with enrichment for the terms 'ribonucleoprotein complex' (*P* =1.03e-29), 'chaperone' (*P* =1.31e-10), and 'mitochondrion' (*P* =1.39e-10) and for the terms 'translational elongation' (*P* =3.58e-73) and 'ribosome' (*P* =2.17e-67), respectively. Then, a community centered on the carbamoyl-phosphate synthetase 2, aspartate transcarbamylase, and dihydroorotase gene (*CAD*) is enriched for 'ncRNA metabolic process' (*P* =2.31e-04).

Subsequently, we performed differential analysis of gene expression data by comparing M3 samples with the other AML samples for each data set. A diagram of differential analysis significance versus connectivity (Figure 1B) shows for each node the number of connections (degree) and the worst (least significant) *P*-value between the two differential analyses accomplished on the two separate datasets. As expected, genes with a high level of connectivity, which are commonly referred to as hubs, show lower significance in terms of differential expression [21]. Exception are represented by a few genes indicated by arrows in Figure 1A,B (*ITGB2*, *LRP1* and *HMOX1*), which show a very high degree of connectivity together with a high level of M3-specific dysregulation, thus emerging as key elements.

Interestingly, the two hubs most de-regulated in APL are both involved in mediating interaction with the ECM and cell migration. Specifically, expression of β2 integrin (ITGB2) has been variously associated with cell motility [22], and is specifically down-regulated on the cell surface of M3 blasts compared with other AMLs [23]. The low-density lipoprotein receptor-related protein 1 (LRP1) is a large receptor whose extracellular domain mediates the binding of various ligands associated with the ECM, and cooperation between LRP1 and ITGB2 was reported to mediate cell adhesion in leukocytes [24]. Similarly to ITGB2, LRP1 has also been variously associated with tumor progression by regulating the balance of adhesion detachment in malignant cells amongst other functions [25]. Finally, the third hub encodes heme oxygenase 1 (HMOX1), an enzyme involved in defense of cells from oxidative stress and that, due to this cytoprotective function, may influence the resistance of cancer cells to pharmacological treatment [26].

### A distinctive subnetwork for acute promyelocytic leukemia

**Figure 2:**
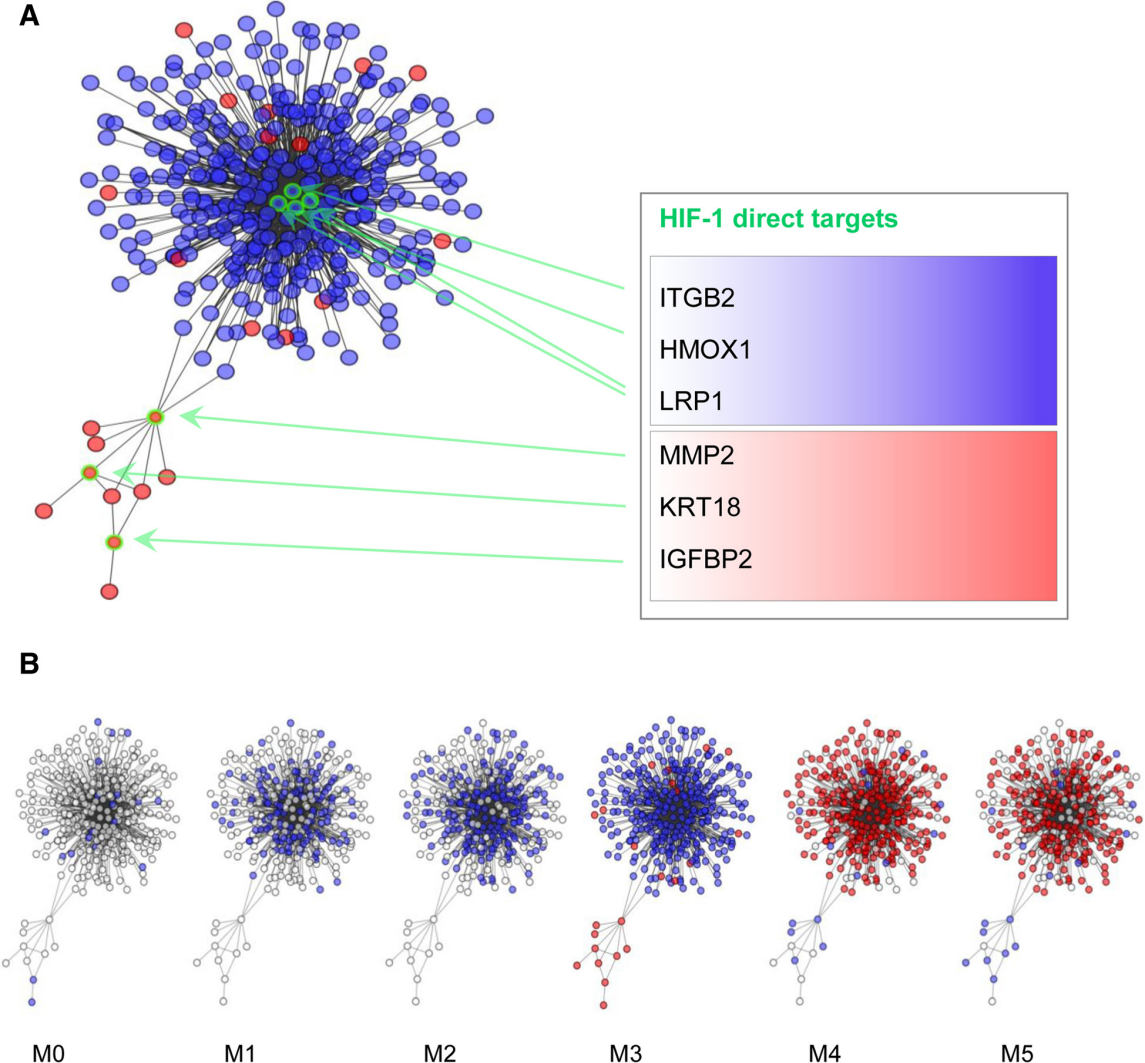
**A HIF-1-dependent subnetwork is specifically dysregulated in APL. (A)** A subnetwork was obtained by extracting from the overall AML network only transcripts that are differentially expressed (FDR adjusted *P*-value <0.05) in the comparison between APL (M3) and other AML subtypes in two AML data sets. Six HIF-1 direct targets are included: MMP2, KRT18 and IGFBP2 in the up-regulated module; and ITGB2, HMOX1 and LRP1 in the vast community that is mostly down-regulated and centered on them. **(B)** The same subnetwork observed across different AML subtypes (from M0 to M5). Down-regulated transcripts are in blue, up-regulated transcripts are in red, non-differentially expressed transcripts are empty circles.

In a further step, we integrated gene expression analysis and network analysis by selecting from the global network only transcripts differentially expressed in APL. We considered only transcripts that were significant (false discovery rate (FDR) adjusted *P*-value <0.05) in both data sets. The subnetwork obtained with this analysis is depicted in Figure 2A (transcripts up-regulated are in red, transcripts down-regulated in blue). Only a few nodes are neglected, which refer to isolated transcripts or small circuits containing at most 10 genes.

Two main modules can be seen in the network: one contains a few up-regulated transcripts, while the other represents a wider group of transcripts that are mostly down-regulated in APL. The part of the network containing down-regulated genes includes the probe sets for *ITGB2*, *HMOX1* and *LRP1*, which were already identified as hubs in the AML network specifically dysregulated in APL with respect to other AML subtypes.

Consistent with down-regulation of a vast group of genes showing overall enrichment for cell adhesion, probe sets corresponding to the highly correlated HIF-1 target genes *MMP2*, *KRT18* and *IGFBP2* are found amongst the nodes of the first group, which are up-regulated in APL with respect to all other AML subtypes (Figure 2A; Additional file 2). Matrix metalloproteinase MMP2 is a degradative enzyme involved in ECM breakdown and tumor growth, metastasis and angiogenesis [27]. The transcripts for MMP2 and the cytoskeletal protein KRT18 display a high level of correlation across all AML samples, and are directly linked (highly dependent) and both connected to the *WT1* and *MLC1* genes in the specific subnetwork identified in APL. The Wilms tumor 1 gene (*WT1*) encodes a transcription factor for which some variants have been already associated with leukemia and cancer [28]; moreover, high *WT1* mRNA levels in the bone marrow of AML patients were associated with poor prognosis [29]. The protein product of *MLC1* is not functionally characterized but sequence analysis suggests a possible role as an integral membrane protein [30].

Insulin-like growth factor binding protein 2 (*IGFBP2*) is a recognized oncogene whose role was shown in several cancer types. Generally, *IGFBP2* expression correlates with disease progression [31]. IGFBP2 can exert IGF-independent functions by interacting with ECM components and integrins. The integrin binding function of IGFBP2 has been shown to be responsible for decreased cell adhesion and increased cell migration in a Ewing’s sarcoma cell line [32]. Moreover, in glioblastoma, IGFBP2 enhances cell invasion by activating MMP2, to which it is highly correlated in both glioblastoma [33] and APL. Finally, it was recently reported that IGFBP2 is over-expressed in APL, where it exerts a key role in mediating survival and migration [34].

The *MMP2* gene appears to play a pivotal role in the subnetwork since it connects the module of the up-regulated genes with the integrin-dependent community (Figure 2), through a direct link with the genes *CPA3*, *TNFRSF4*, *CDC42EP3*, *MAP1A* and *LILRA2*. These genes are all down-regulated in APL, like the vast majority of the community to which they belong. Among them, *CDC42EP3* and *MAP1A* are genes known to participate in cytoskeleton organization, with CDC42EP3 being involved in actin cytoskeleton remodeling during changes in cell shape [35], and MAP1A being associated with microtubule assembly [36].

Overall, by integrating differential analyses of AML samples it emerges that a distinctive subnetwork of HIF target genes is peculiarly dysregulated in APL, with genes involved in invasion up-regulated and genes key for adhesion concordantly down-regulated. The specific modulation of these genes in APL shows characters typical of EMT, which is strongly associated with the pathogenesis of solid tumors, and only recently is beginning to be implicated in leukemia [37].

EMT is a fundamental process during embryogenesis characterized by a loss of cell-cell adhesion, anchorage to the substrate and apical-basal polarity, and increased cell motility [38]. EMT is also a crucial aspect in tumor progression: it confers on cancer cells migration properties and invasiveness, which lead to metastasis formation [39].

Several transcription factors have been implicated in EMT, with HIF-1 included among them since a number of direct HIF-1 target genes are involved in regulating cell motility, cytoskeletal organization and ECM metabolism [40]. Thus, we examined the expression of the main EMT regulators across the AML subtypes. We found a striking up-regulation of *TWIST1* expression in APL compared with all other subtypes (FDR adjusted *P* <0.0018), although limited to the TCGA dataset. Given the recognized role of this transcription factor in EMT, we decided to also consider this gene in the validation phase.

### The APL-specific subnetwork is gradually dysregulated across different AMLs

We next analyzed the APL-specific subnetwork across different FAB subtypes to study its behavior following progressive differentiation states of leukemic cells. In Figure 2B, transcripts are depicted as empty circles if they were not differentially expressed in the comparison between each AML subtype and the other AMLs, while they are filled in blue or red, if they are down- or up-regulated, respectively. As apparent, this topologically characterized gene signature shows very specific behavior from M0 to M5 (from minimally to highly differentiated leukemic cells). The up-regulation of *MMP2*, *KRT18*, *IGFBP2* and their neighbor genes is exclusive to M3, and the crowded group of genes related to β2 integrin shows a clear trend from absence of dysregulation in M0 to progressive down-regulation up to M3. Afterwards, a clear switch takes place from M3 to M4, involving the whole subnetwork. The specific behavior of these genes can be appreciated also in the heatmap of the clustered profiles (Additional file 3), which also shows a clear segregation of M3 samples. This analysis suggests that dysegulation of genes related to EMT-like characteristics and cell migration downstream of HIF-1 is not a general phenomenon of leukemic transformation within the myeloid lineage but rather segregates with specific leukemia subtypes.

### Validation

To validate that the HIF-1 target genes identified by our study are regulated by HIF-1 also in the hematopoietic context, HIF-1α was silenced in APL-derived NB4 cells and expression of *MMP2*, *KRT18*, *IGFBP2*, *ITGB2*, *HMOX1*, and *LRP1* was assessed by RT-PCR. Expression levels of all genes except *KRT18* was significantly affected by HIF-1α down-regulation in NB4 cells (Additional file 4). Moreover, all genes except *KRT18* were significantly up-regulated in NB4 cells upon exposure to hypoxia (Additional file 5).

The module of dysregulated genes specific to APL was identified by processing two independent gene expression data sets, retaining only significantly differentially expressed genes found in both. To validate the results obtained with the microarray data, we assessed the expression for the six key genes of the subnetwork along with *TWIST1* in RNA-Seq data available for 178 out of the same 197 patients of the TCGA microarray data set. As reported in Additional file 6, we confirmed the differential expression of these genes in M3 compared with other FAB subtypes.

Finally, we assessed by RT-PCR the expression of the same genes in bone marrow samples from AML patients of IRCCS Ospedale San Raffaele. As representative samples, we selected four patients with M3 and four patients with M5, as these two leukemias represented opposite patterns of expression of the genes of the subnetwork. In addition, we also validated differential expression of *TWIST1*. The comparison between M3 and M5 (Additional file 7) confirmed the dysregulation trend observed in the two main cohorts of AML patients for the key genes of the subnetwork and also for the EMT regulator TWIST1.

### The acute promyelocytic leukemia subnetwork discriminates leukemic promyelocytes from their normal counterpart

**Figure 3:**
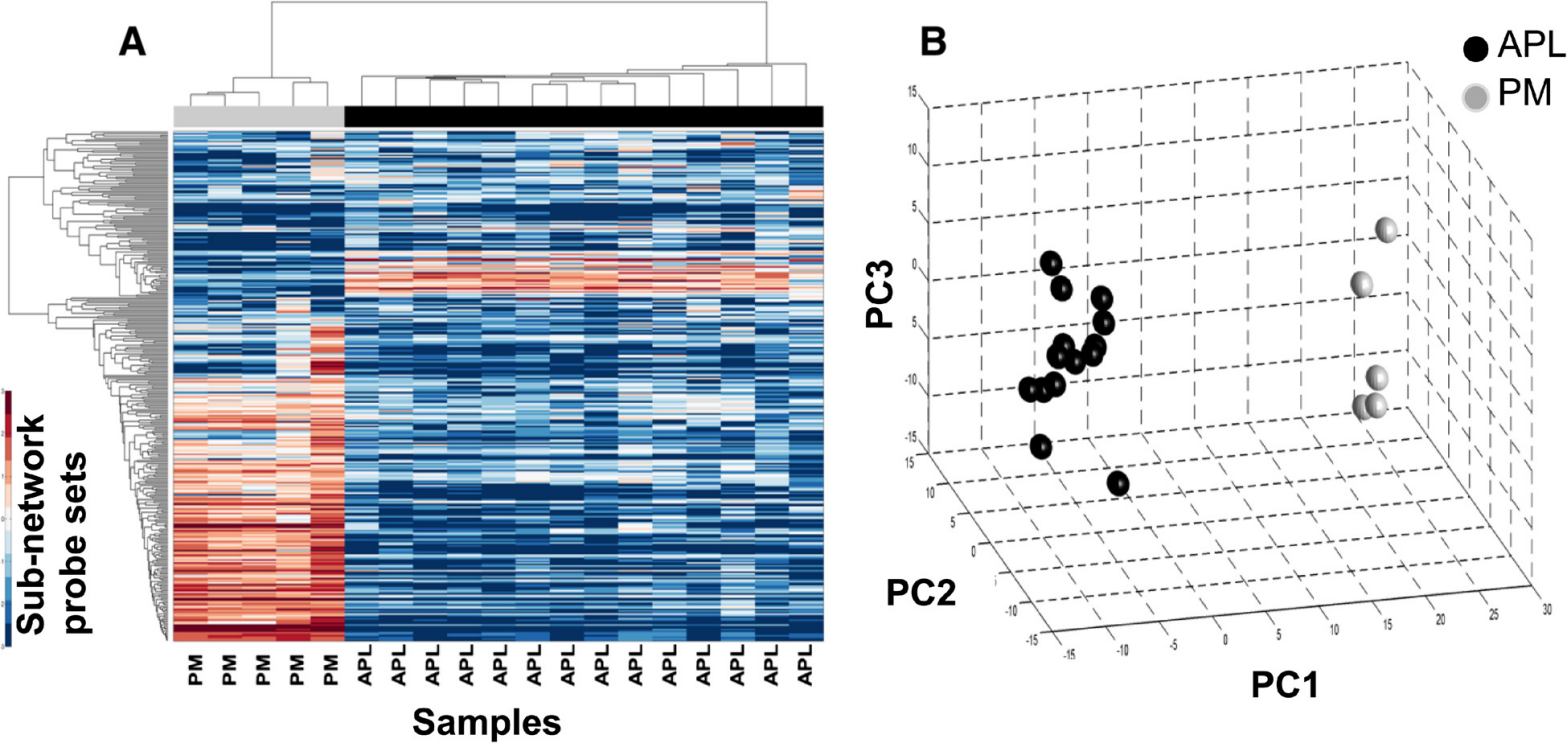
**The APL-specific HIF-1-dependent subnetwork distinguishes leukemic promyelocytes from their normal counterpart. (A)** Unsupervised clustering shows a clear segregation between normal (PM, n =5, grey bar cluster) and leukemic promyelocytes (APL, n =14, black bar cluster) samples. **(B)** Comparison of the HIF-dependent subnetwork in leukemic versus normal promyelocytes through principal component analysis.

To assess if the differential expression of the genes of the APL subnetwork is dependent on the specific developmental stage of affected cells, we analyzed their values in a dataset of samples comprising leukemic promyelocytes (APL, n =14) and their normal counterpart (PM, n =5) [41]. Profiles were extracted for the 329 transcripts (corresponding to 246 genes) present in the M3-specific subnetwork. Unsupervised clustering of expression profiles (Figure 3A) shows a clear segregation of PM samples from APL samples, which is confirmed by principal component analysis (Figure 3B). These data clearly indicate that dysregulated expression of these genes when compared with other AML subtypes is not caused by their expression levels in normal promyelocytes, but is indeed specific of APL promyelocytes.

**Figure 4:**
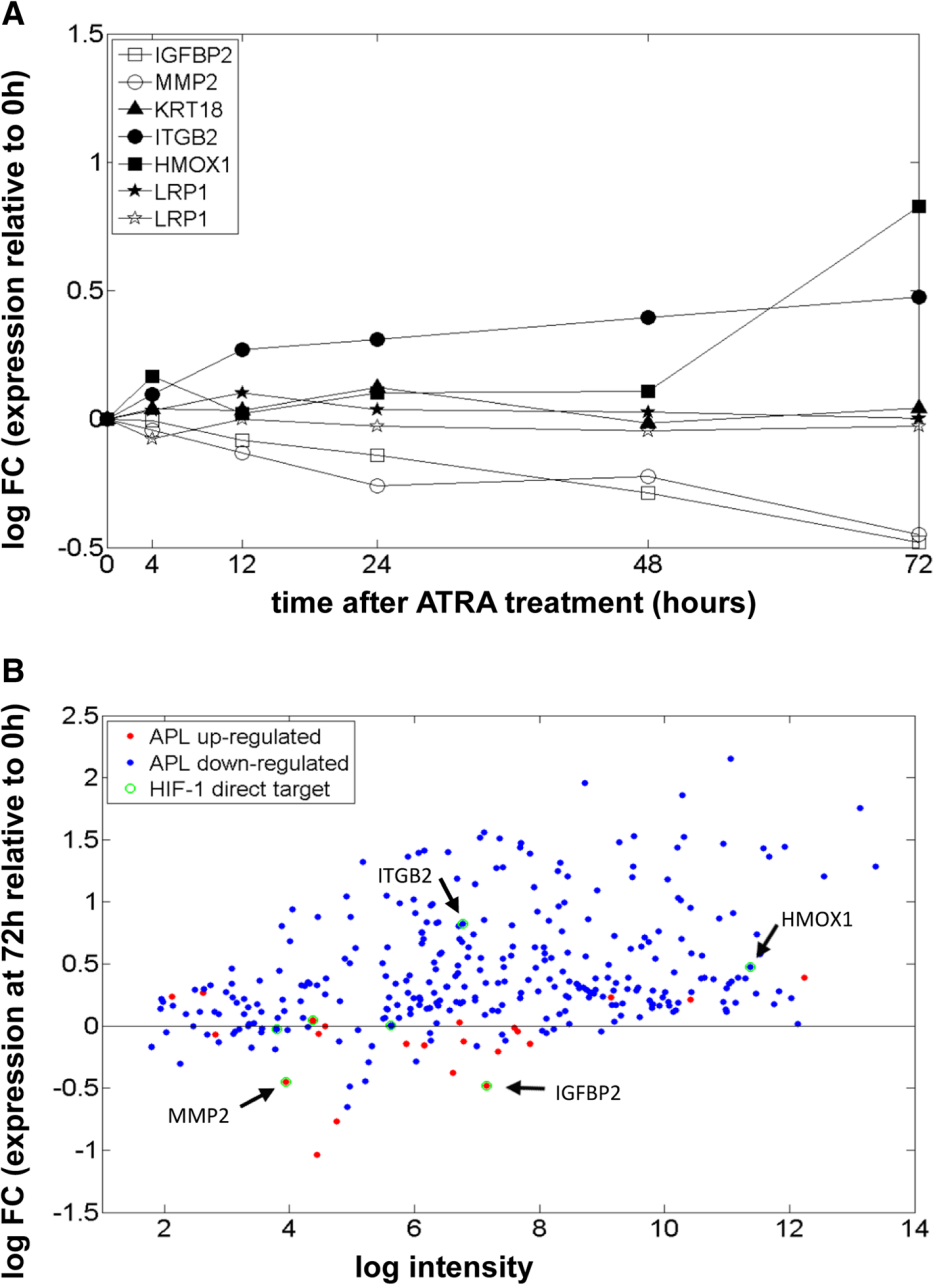
**Response of the subnetwork genes to ATRA treatment. (A)** mRNA expression profiles of a time-course experiment in NB4 cell lines after ATRA administration are reported; values are relative to the time of drug administration (0 h), empty symbols indicate genes that are up-regulated in APL, filled symbols genes that are down-regulated. Reversal of APL-specific dysregulation is observed for four genes: *IGFBP2* and *MMP2* show decreased expression whereas *ITGB2* and *HMOX1* show decreased expression upon ATRA treatment. **(B)** Overall behavior of the whole subnetwork (APL down-regulated transcripts in blue, APL up-regulated transcripts in red), observed 72 hours after treatment, highlights a general inversion of APL dysregulation. FC, fold change.

We then assessed the expression of the HIF-1 target genes belonging to the subnetwork in NB4 cells treated with all-*trans* retinoic acid (ATRA) at different time points [42]. ATRA treatment provoked the reversal of gene expression changes observed in APL for *ITGB2*, *HMOX1*, *IGFBP2* and *MMP2* (Figure 4A). Also, looking at the overall subnetwork at the last time point (72 h) of the experiment (Figure 4B), the vast majority of APL down-regulated genes (in blue) show a positive fold-change relative to the absence of treatment and, conversely, most APL up-regulated genes (in red) show a decrease in expression after treatment.

Taken together, these data indicate that although differential expression of the HIF-related network in APL versus other AML subtypes is not caused by the different cells of origin (promyelocytes versus other types of myeloid cells), nonetheless when promyelocytes are induced to differentiate by ATRA treatment the expression of these genes is significantly affected, thus suggesting that this subnetwork may play an important role in the processes of leukemogenesis or leukemia maintenance.

### microRNAs of the miR-181 family are the most correlated with the down-regulated genes in the APL module

**Figure 5:**
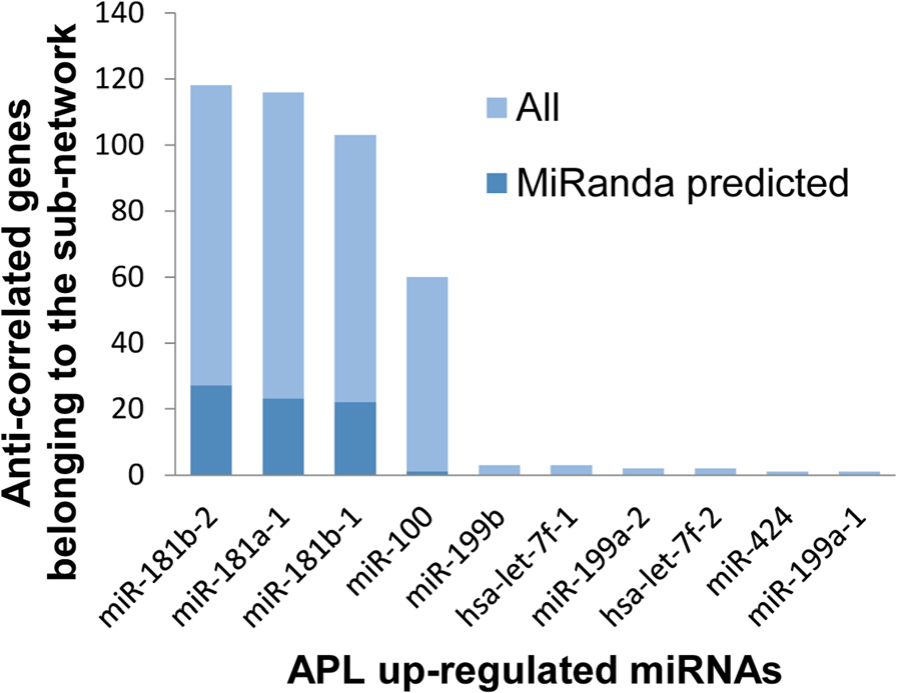
**microRNAs of the miR-181 family have the highest number of anti-correlated genes in the APL subnetwork.** microRNAs are listed according to the number of anti-correlated genes (Pearson correlation coefficient less than −0.4) found in the APL subnetwork. All the microRNAs reported are significantly up-regulated in APL (Bonferroni corrected *P*-value <0.05). The proportion of potential direct targets predicted by MiRanda is indicated for each microRNA.

Because the subnetwork that we have identified includes a large community of down-regulated genes (the β2 integrin-dependent group), we searched for microRNAs that can potentially target these genes. To do this, we exploited microRNA expression data available for the samples of the second data set (from TCGA Data Portal). We identified the microRNAs significantly (Bonferroni corrected *P*-value <0.05) up-regulated in M3 and showing a correlation coefficient lower than −0.4 with respect to at least one transcript belonging to the β2 integrin gene group. A list of 10 microRNAs was generated in this way. They are ordered in Figure 5 according to the number of anti-correlated transcripts belonging to the down-regulated part of the APL subnetwork; these anti-correlated transcripts are significantly enriched for the SP_PIR keyword 'cell adhesion' (Bonferroni corrected *P*-value =5.41e-04). For each microRNA, the proportion of transcripts that are predicted as direct targets by MiRanda (that is, with the recognition of a potential biding site) is also reported. It is worth noting that among the selected microRNAs, miR-100 was already found up-regulated in pediatric APL [43] and miR-424 is considered as a 'hypoxamir', being implicated in oxygen-dependent changes by stabilizing HIF-1α and increasing transcription of its target genes. It is up-regulated in cells during angiogenesis, vascular remodeling, invasion and proliferation [44].

More importantly, the microRNAs with the highest amount of potential targets belong to the miR-181 family, which was very recently found to promote EMT in ovarian cancer progression [45].

## Conclusions

By applying an integrated approach to study the contribution of HIF-1 signaling to APL, we have identified a gene subnetwork that distinguishes APL from other AML subtypes; it is centered around genes whose transcription is hypoxia inducible and are further modulated in APL unveiling characteristic of EMT gene expression programs.

Starting from the known direct targets of HIF-1, we constructed a downstream regulatory network depicting the contribution of this master regulator of hypoxia adaptation to the APL transcriptional signature. Gene expression profiles from two independent AML data sets derived from patient samples were processed: to enforce the prediction, only interactions and changes in expression confirmed in both datasets were retained. The network module that was identified shows overexpression of genes linked to migration and invasiveness (for example, *KRT18* and *MMP2*) along with down-regulation of a vast community of genes whose main hub is the integrin *ITGB2* gene, showing an overall enrichment for genes regulating cell adhesion. Altogether, dysregulation of this gene network in APL implicates the involvement of EMT-like processes in this specific leukemia subtype. Accordingly, microRNAs previously implicated in EMT and metastasis in solid tumors, such as those belonging to the miR-181 family, are also dysregulated in APL, possibly participating in the down-regulation of cell adhesion genes.

The process of hematopoietic and leukemic cell migration from bone marrow to peripheral blood and other tissues is not fully elucidated, but according to our data, it appears to play an important role in the development or progression of APL. Our results show that processes occurring during EMT in solid tumors could also be implicated in the pathophysiology of some types of leukemia downstream of HIF-1. Interestingly, within AML subtypes, dysregulation towards EMT-like characteristics appears to be particularly relevant to APL development or progression and, in the future, it will be interesting to elucidate the molecular details of this regulation.

## Additional files

Additional file 1:
**List of the 119 HIF-1 direct targets used in this study.** Genes were selected for presence of functional hypoxia responsive elements (HREs) in their promoters validated by HIF-1 chromatin immunoprecipitation and experimental evidence of hypoxia-dependent transcriptional induction. Gene symbol, aliases, gene ID, gene name and reference PubMed ID are reported for each gene. Additional file 2:
**Scatter plots reporting pairwise correlations between HIF-1 target genes included in the up-regulated module of the APL subnetwork.** **(A)** Correlation between *KRT18* and *MMP2*. **(B)** Correlation between *IGFBP2* and *MMP2*. **(C)** Correlation between *IGFBP2* and *KRT18*. Correlations are computed from the TCGA gene expression data set as Pearson’s correlation coefficients (r) and *P*-values (p) represent the statistical significance of the dependence testing the null hypothesis of no correlation. Additional file 3:
**Unsupervised hierarchical clustering of the expression values of the transcripts belonging to the APL HIF subnetwork.** The heatmap shows the mean-centered log2 expression values of probe sets (rows) across the AML samples (columns) of the TCGA dataset. APL samples segregate in a specific cluster indicated by the black bar. Additional file 4:
**HIF-1α silencing.** Real-time PCR analysis of *MMP2,* *KRT18,* *IGFBP2,* *ITGB2,* *HMOX1* and *LRP1* upon HIF-1α silencing in NB4 cells. Data represent mean values (± standard error of the mean) of three independent experiments. N.s., not significant. Additional file 5:
**Functional relationship between the APL subnetwork and hypoxia.** Real-time PCR analysis of *MMP2*, *KRT18*, *IGFBP2*, *ITGB2*, *HMOX1*, and *LRP1* in NB4 cells cultured in hypoxic conditions for different time points. Data represent mean values (±standard error of the mean) of three technical replicates. Significance is shown at 16 h compared with normoxia (0 h). N.s., not significant. Additional file 6:
**Confirmation of APL subnetwork dysregulation using RNA-Seq data.** The comparison between M3 samples (n =16) and other subtypes (n =162) is reported for *MMP2*, *KRT18*, *IGFBP2*, *ITGB2*, *HMOX1*, *LRP1* and *TWIST1*. Data are expressed as RPKM. Additional file 7:
**Real-time PCR analysis of** ***MMP2*** **,** ***KRT18,*** ***IGFBP2,*** ***TGB2,*** ***HMOX1,*** ***LRP1*** **and** ***TWIST1.*** Expression values in bone marrow samples from M3 (n =4) and M5 (n =4) patients. Differential expression is confirmed for all genes of the subnetwork and for *TWIST1*.

## Abbreviations

AML: acute myeloid leukemia
APL: acute promyelocytic leukemia
ATRA: all-*trans* retinoic acid
ECM: extracellular matrix
EMT: epithelial-mesenchymal transition
FAB: French American British
FDR: false discovery rate
GEO: Gene Expression Omnibus
HIF: hypoxia-inducible factor
IGF: insulin-like growth factor
PM: promyelocyte
RPKM: reads per kilobase per million mapped reads
TCGA: The Cancer Genome Atlas
