# Supplementary figures and images for: A HIF-1 network reveals characteristics of epithelial-mesenchymal transition in acute promyelocytic leukemia
Source: Genome Med. 2014 Dec 1;6(12):84. doi: 10.1186/s13073-014-0084-4 (PMC4249615; doi:10.1186/s13073-014-0084-4)

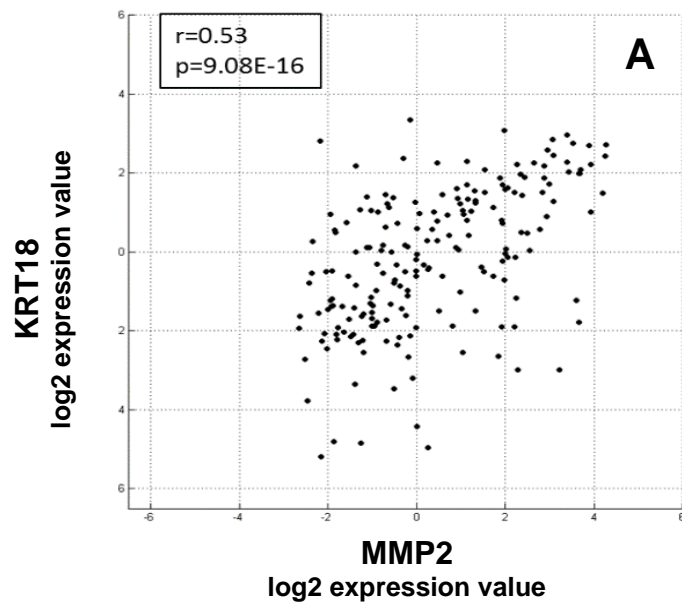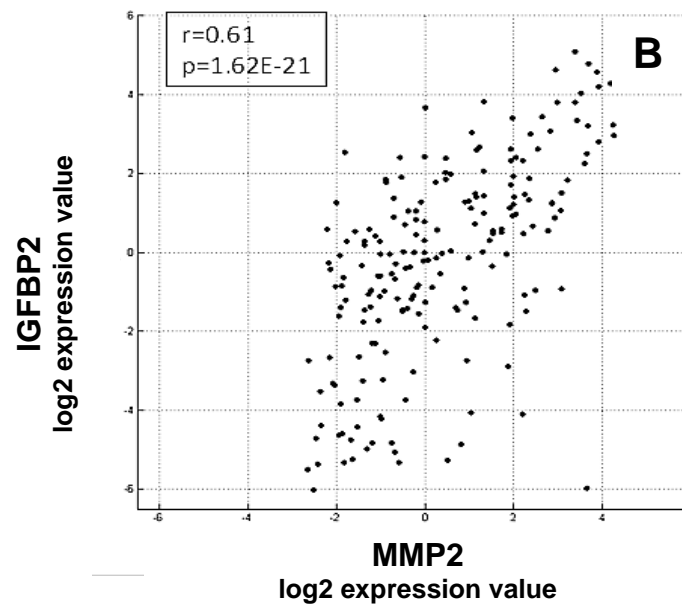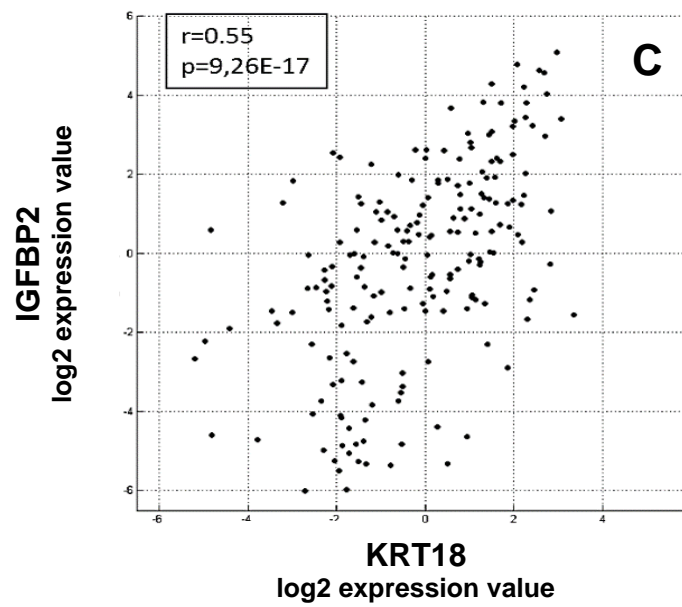

Supplement: Additional file 2: — Scatter plots reporting pairwise correlations between HIF-1 target genes included in the up-regulated module of the APL subnetwork. (A) Correlation between KRT18 and MMP2. (B) Correlation between IGFBP2 and MMP2. (C) Correlation between IGFBP2 and KRT18. Correlations are computed from the TCGA gene expression data set as Pearson’s correlation coefficients (r) and P-values (p) represent the statistical significance of the dependence testing the null hypothesis of no correlation. [file 13073_2014_84_MOESM2_ESM.pdf]

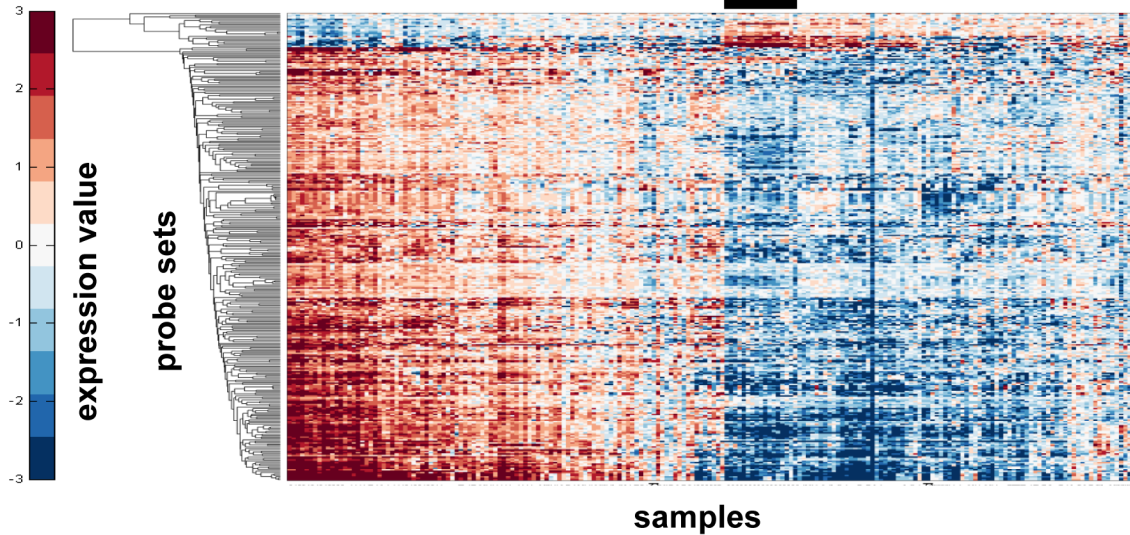

Supplement: Additional file 3: — Unsupervised hierarchical clustering of the expression values of the transcripts belonging to the APL HIF subnetwork. The heatmap shows the mean-centered log2 expression values of probe sets (rows) across the AML samples (columns) of the TCGA dataset. APL samples segregate in a specific cluster indicated by the black bar. [file 13073_2014_84_MOESM3_ESM.pdf]

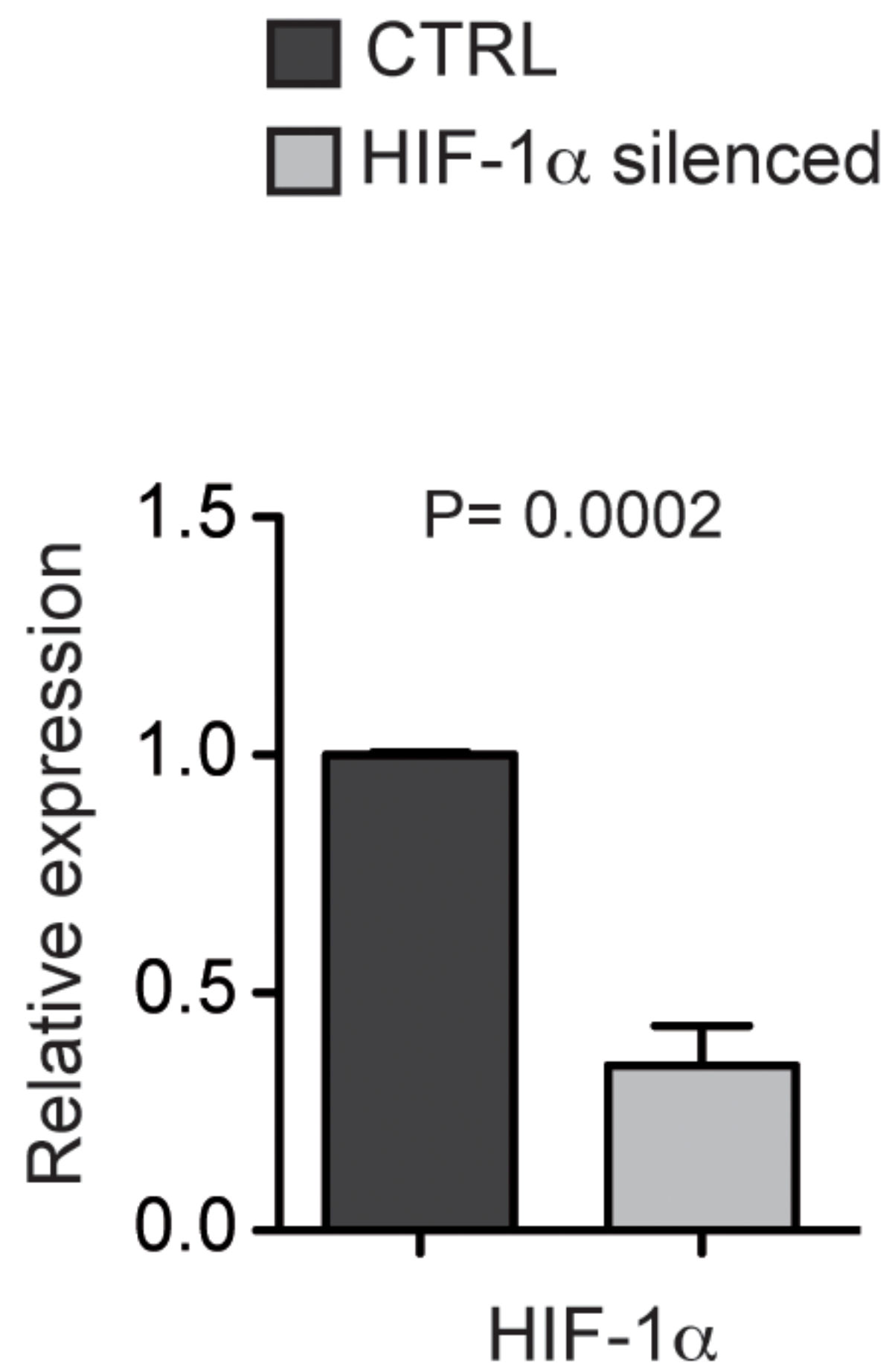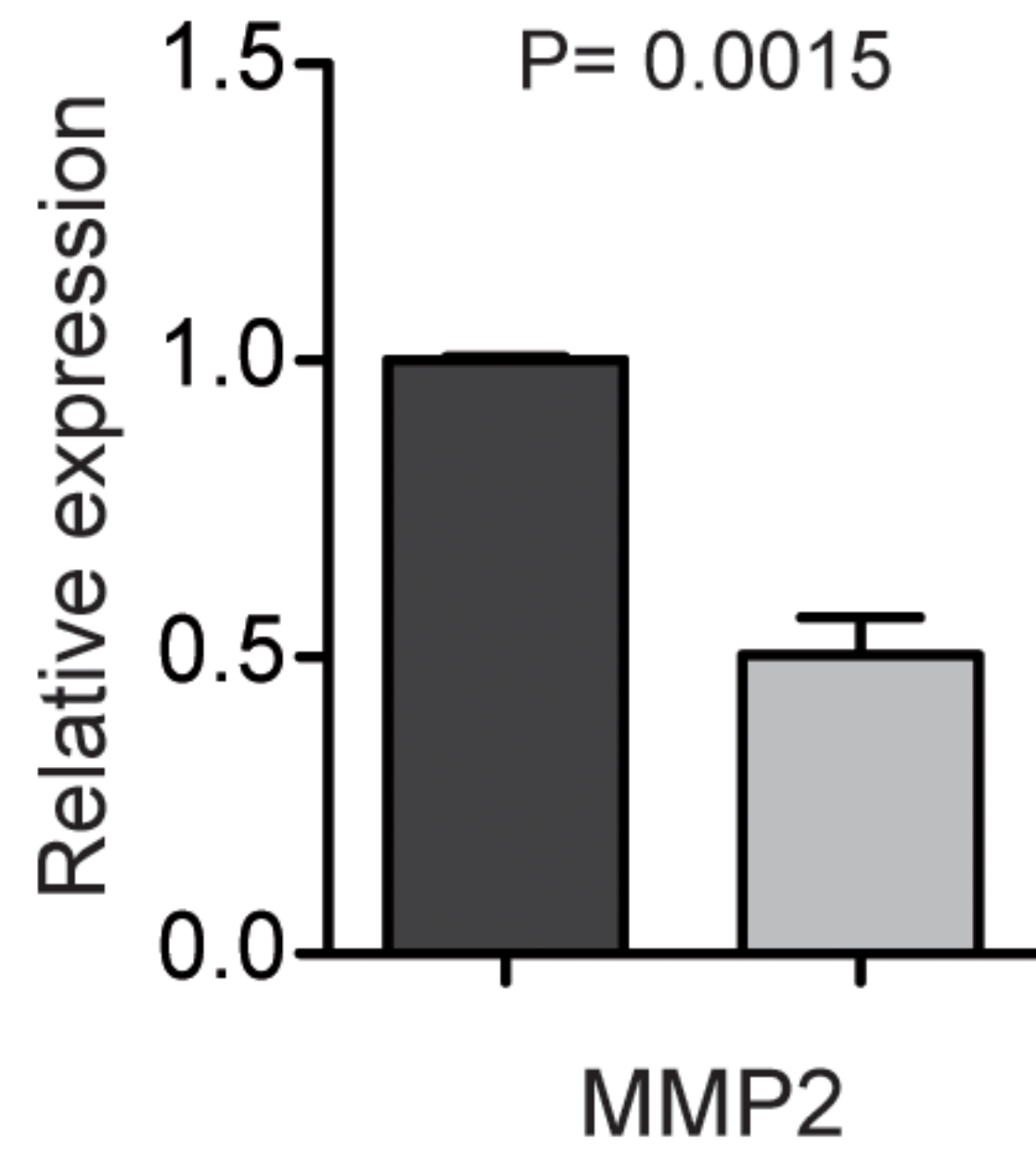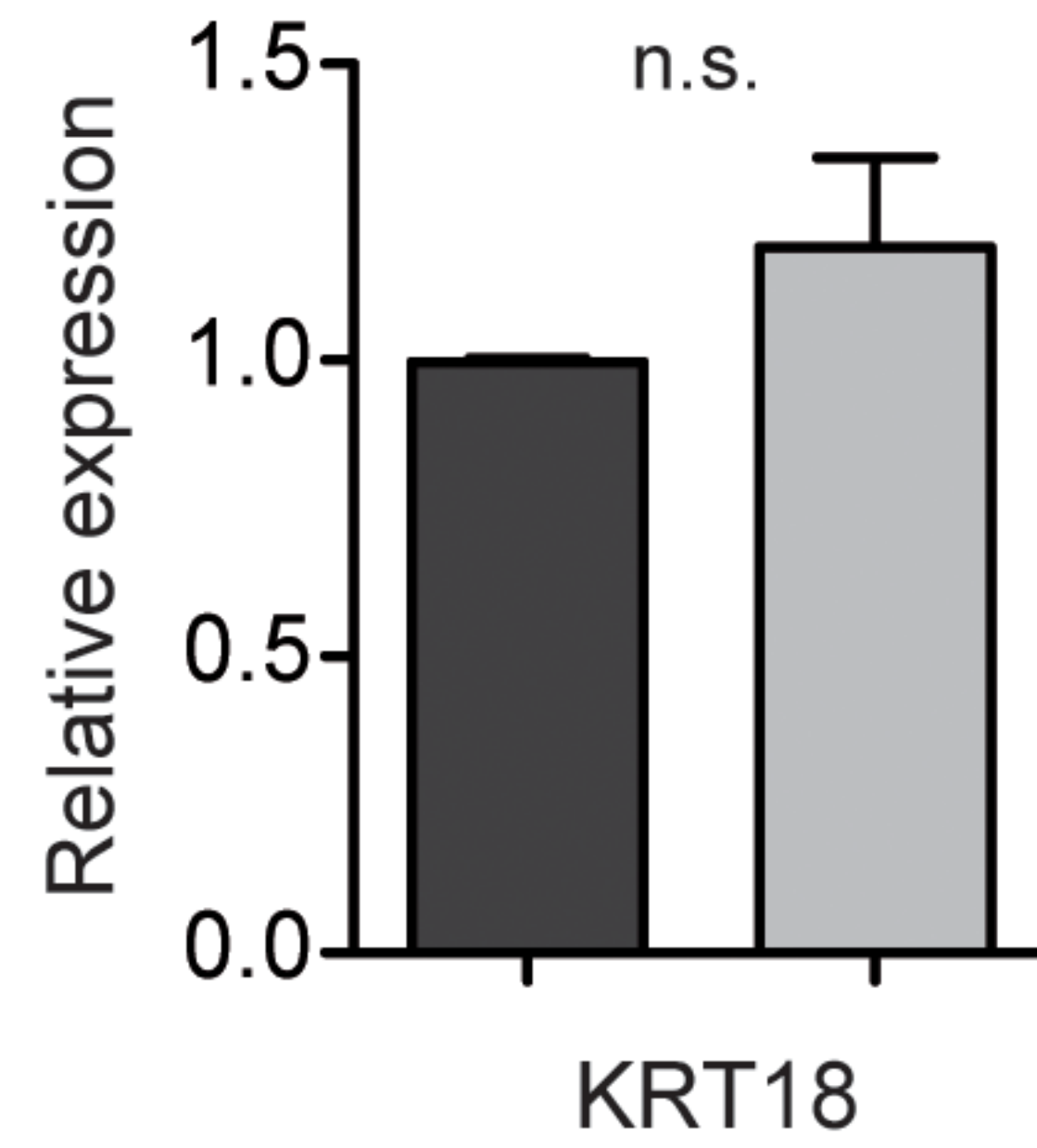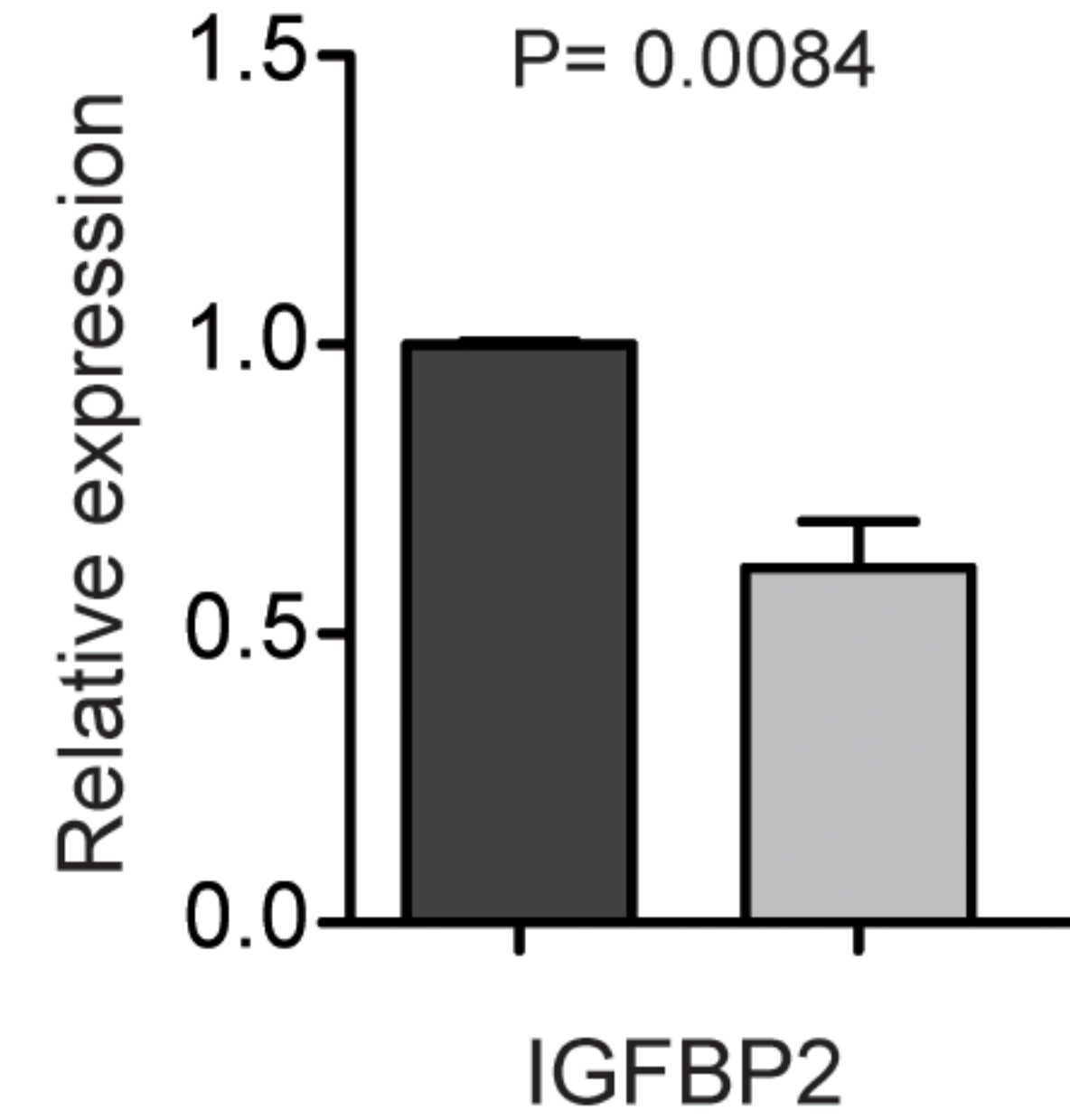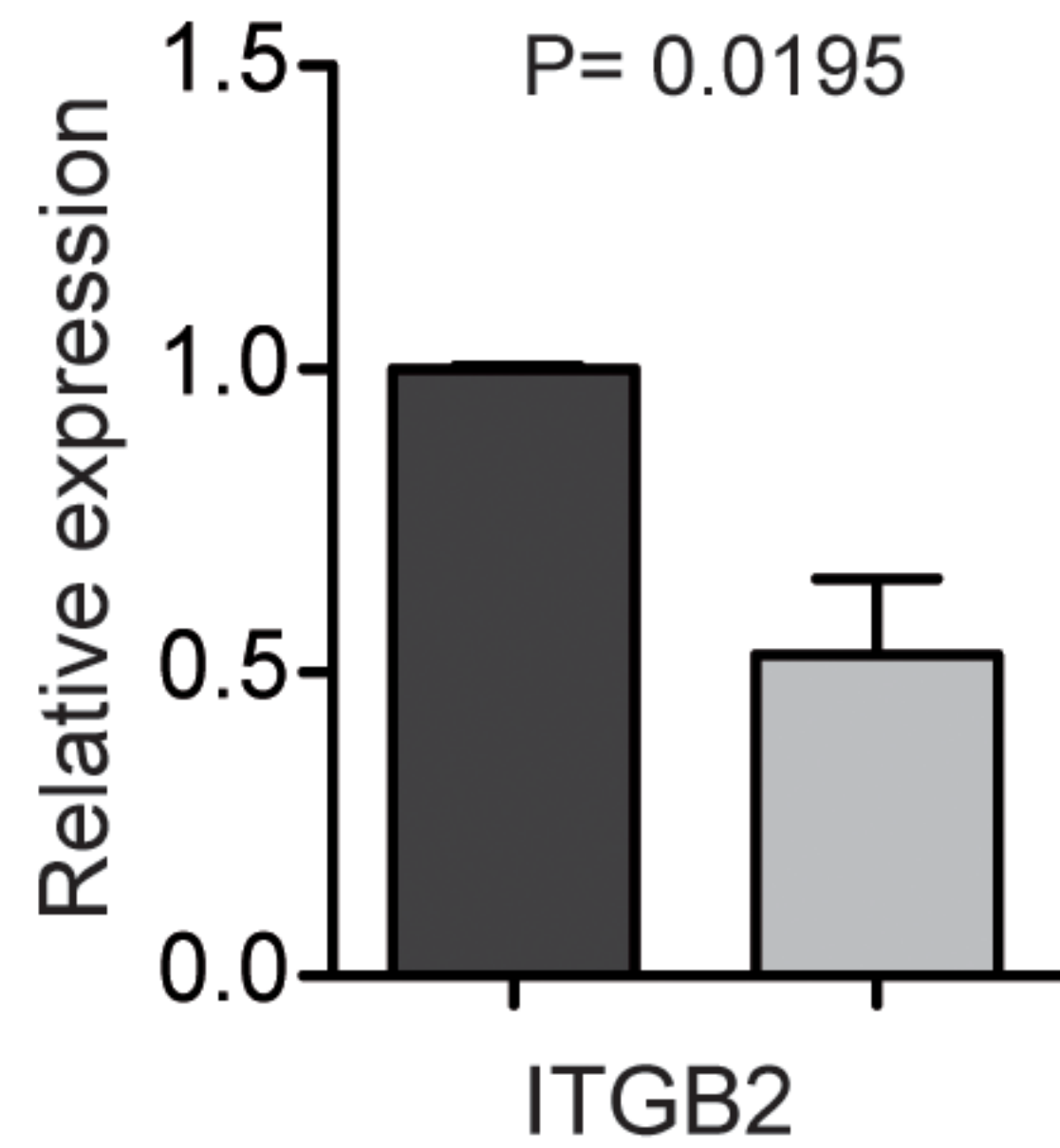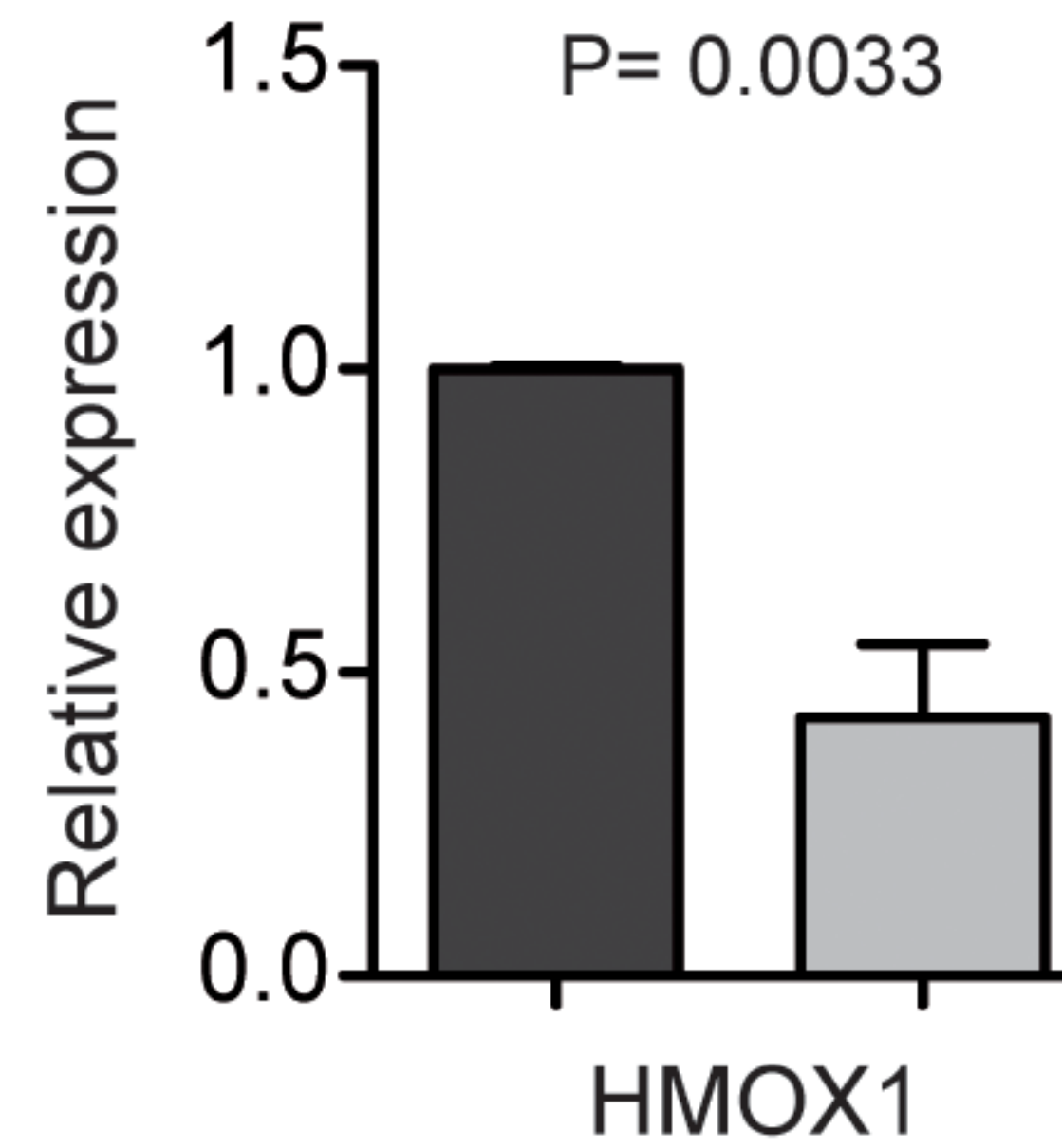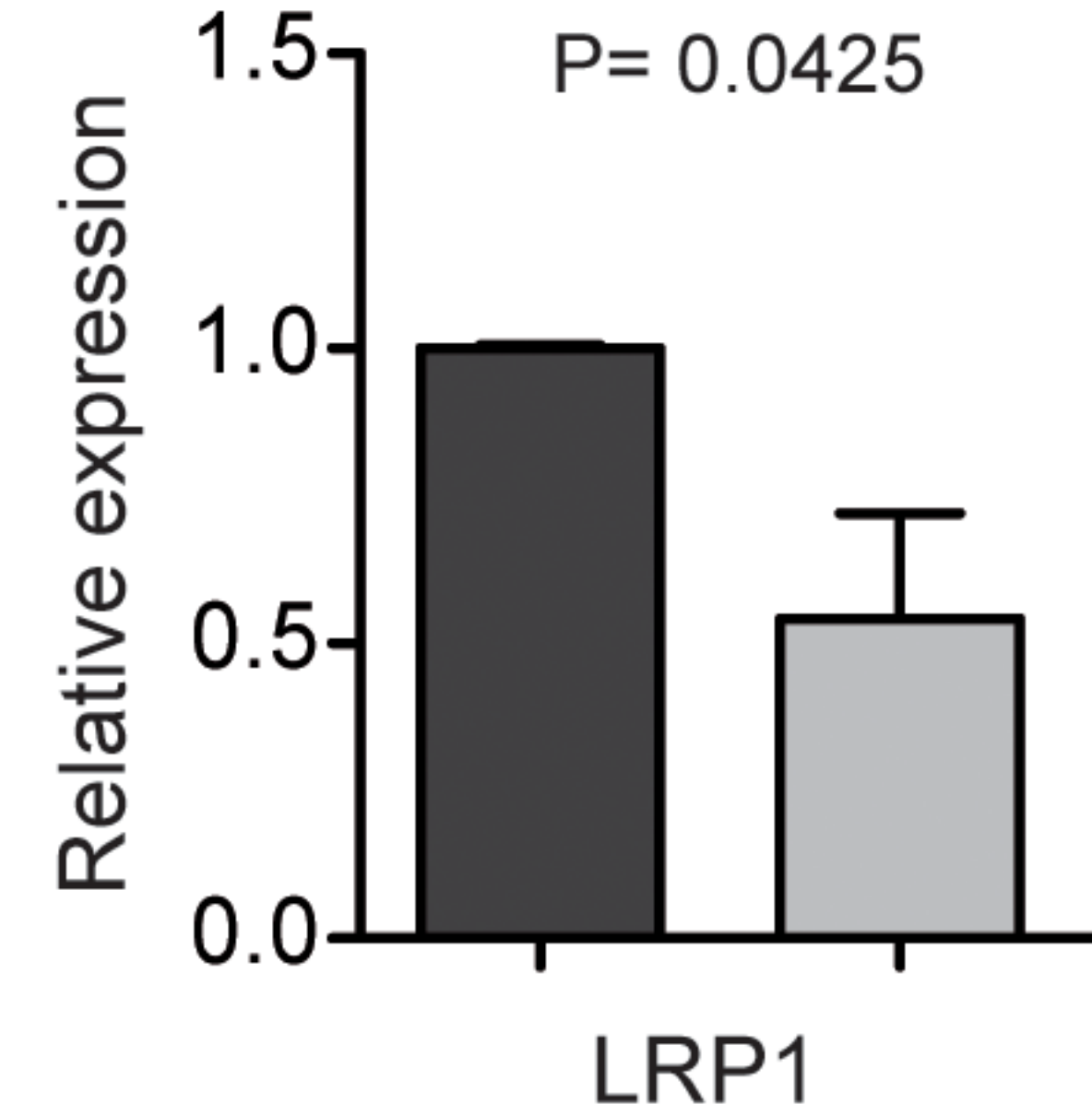

Supplement: Additional file 4: — HIF-1α silencing. Real-time PCR analysis of MMP2, KRT18, IGFBP2, ITGB2, HMOX1 and LRP1 upon HIF-1α silencing in NB4 cells. Data represent mean values (± standard error of the mean) of three independent experiments. N.s., not significant. [file 13073_2014_84_MOESM4_ESM.pdf]

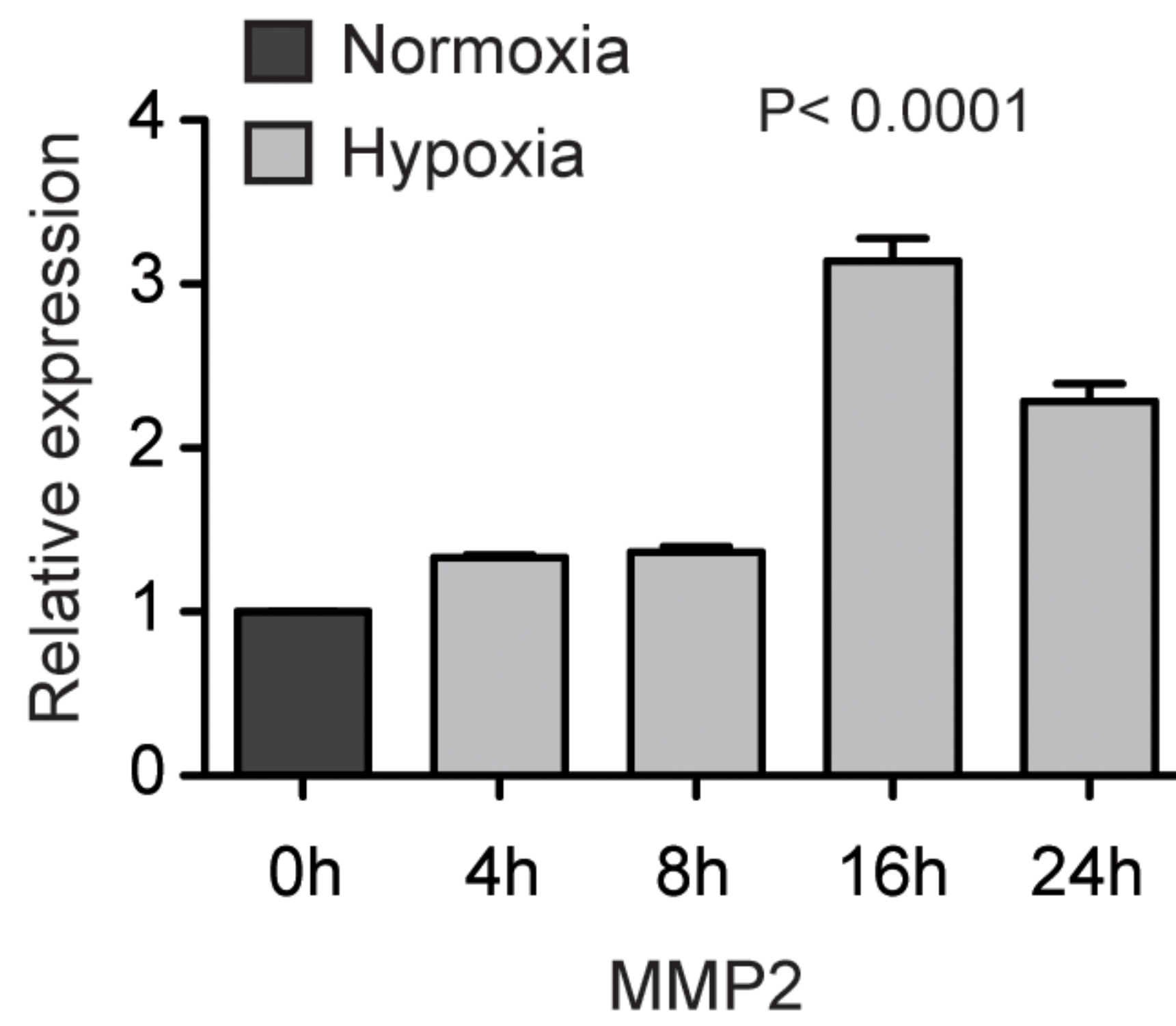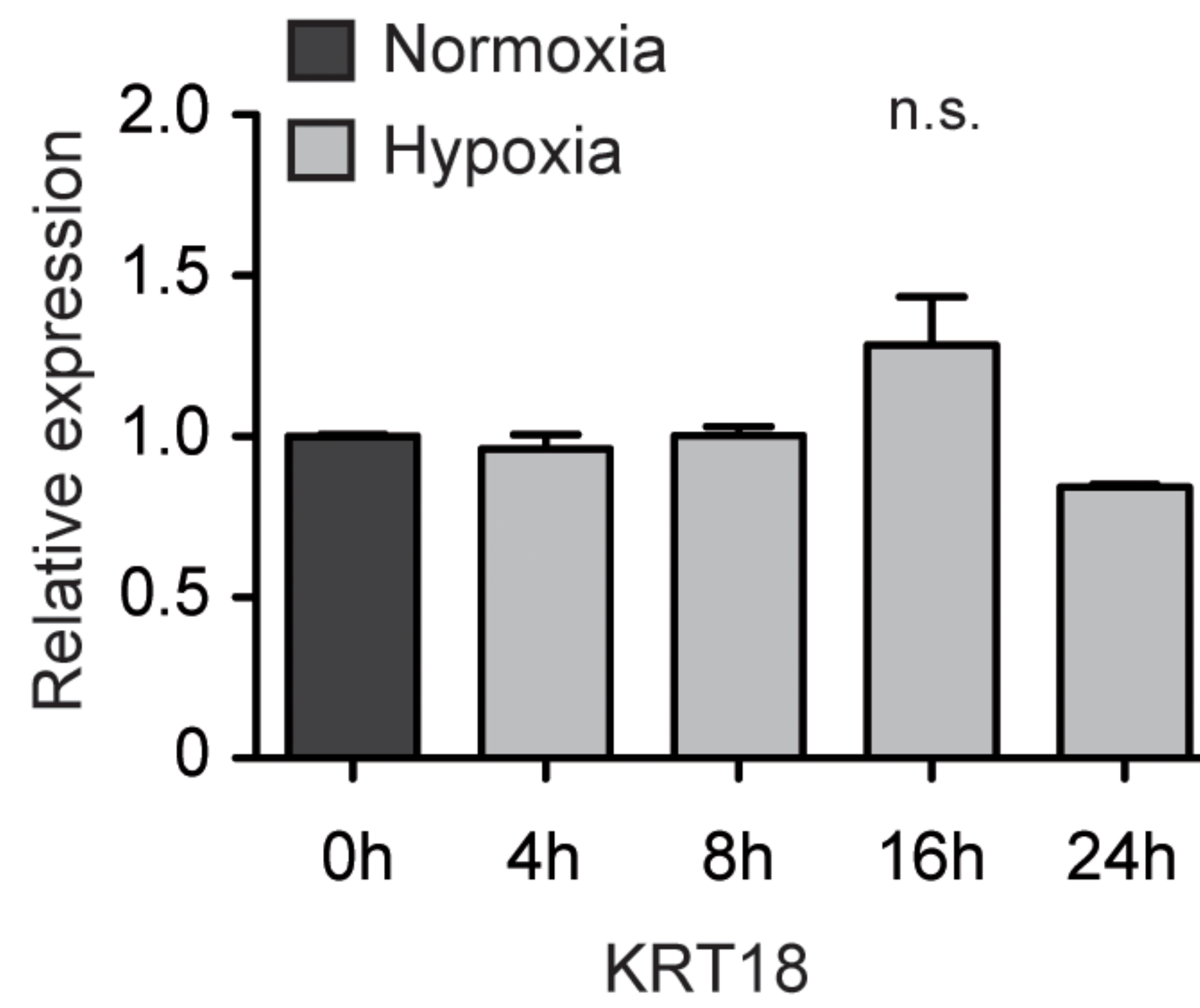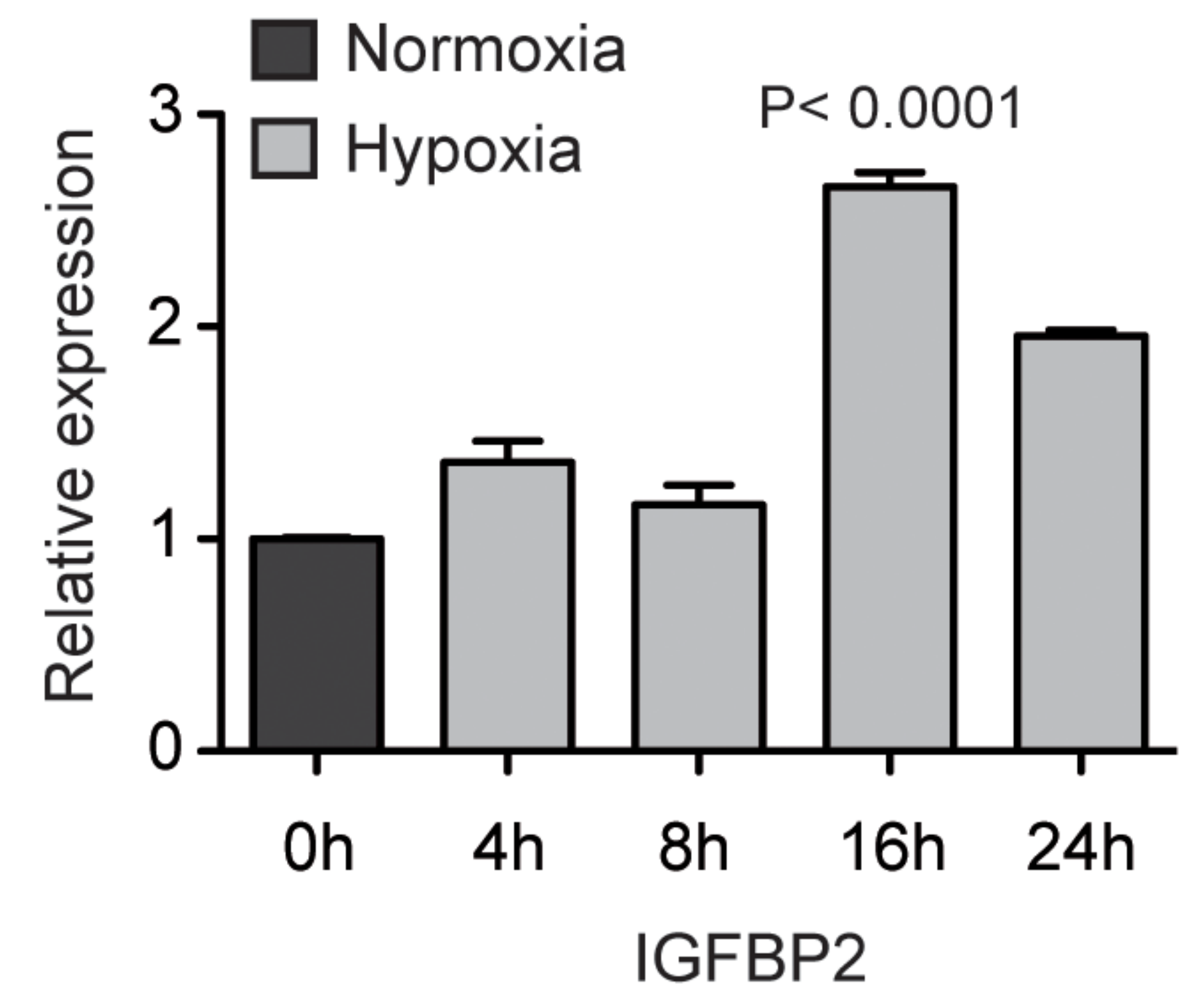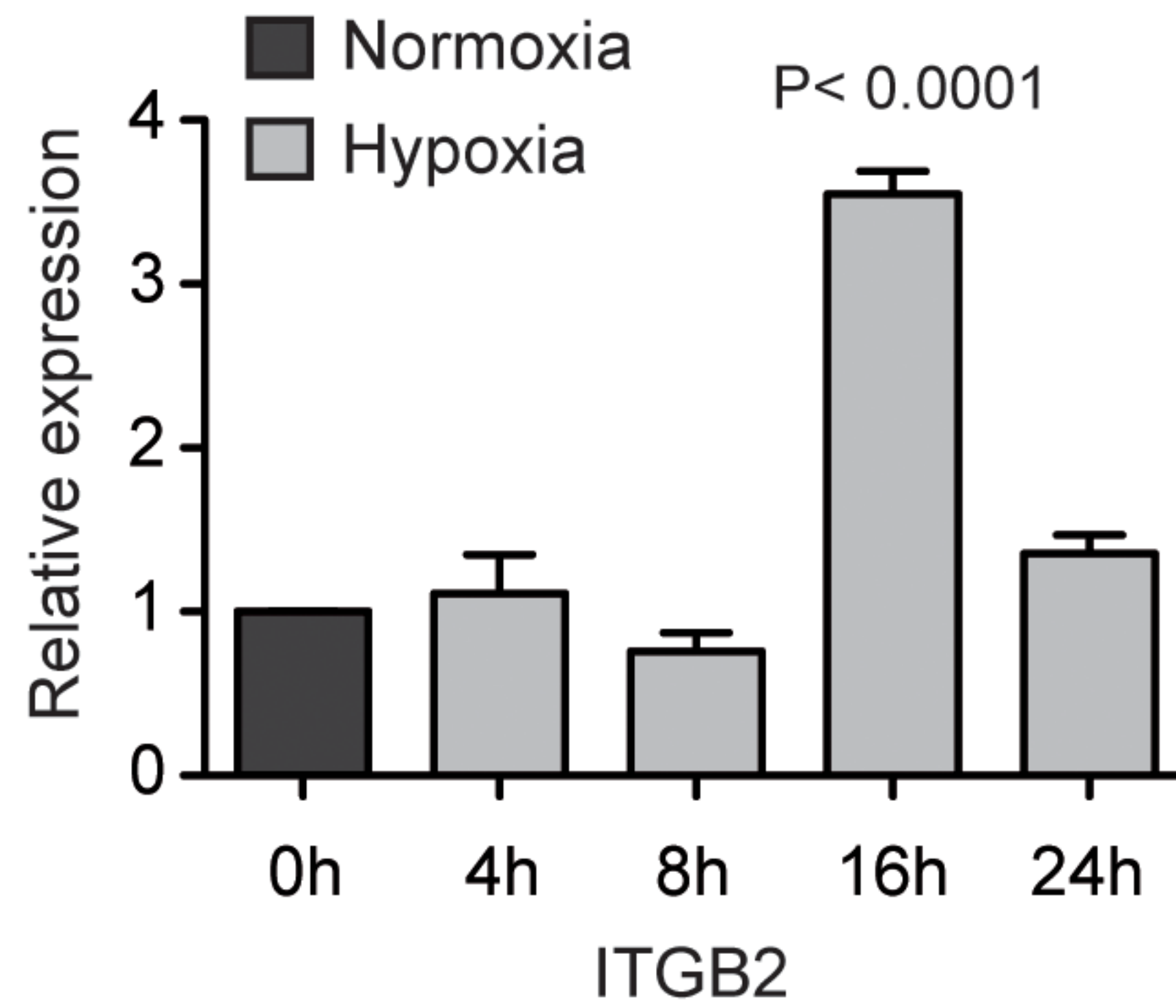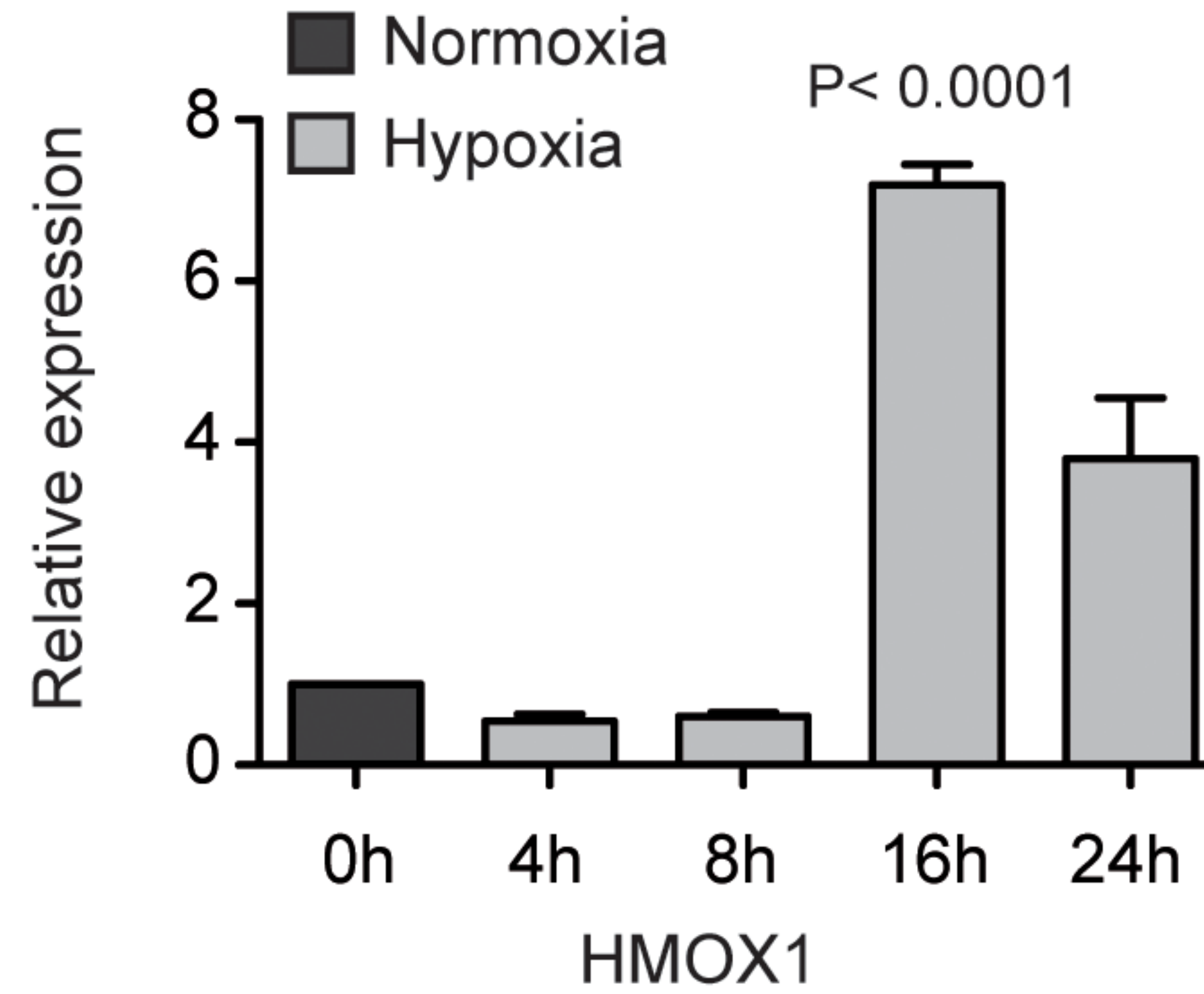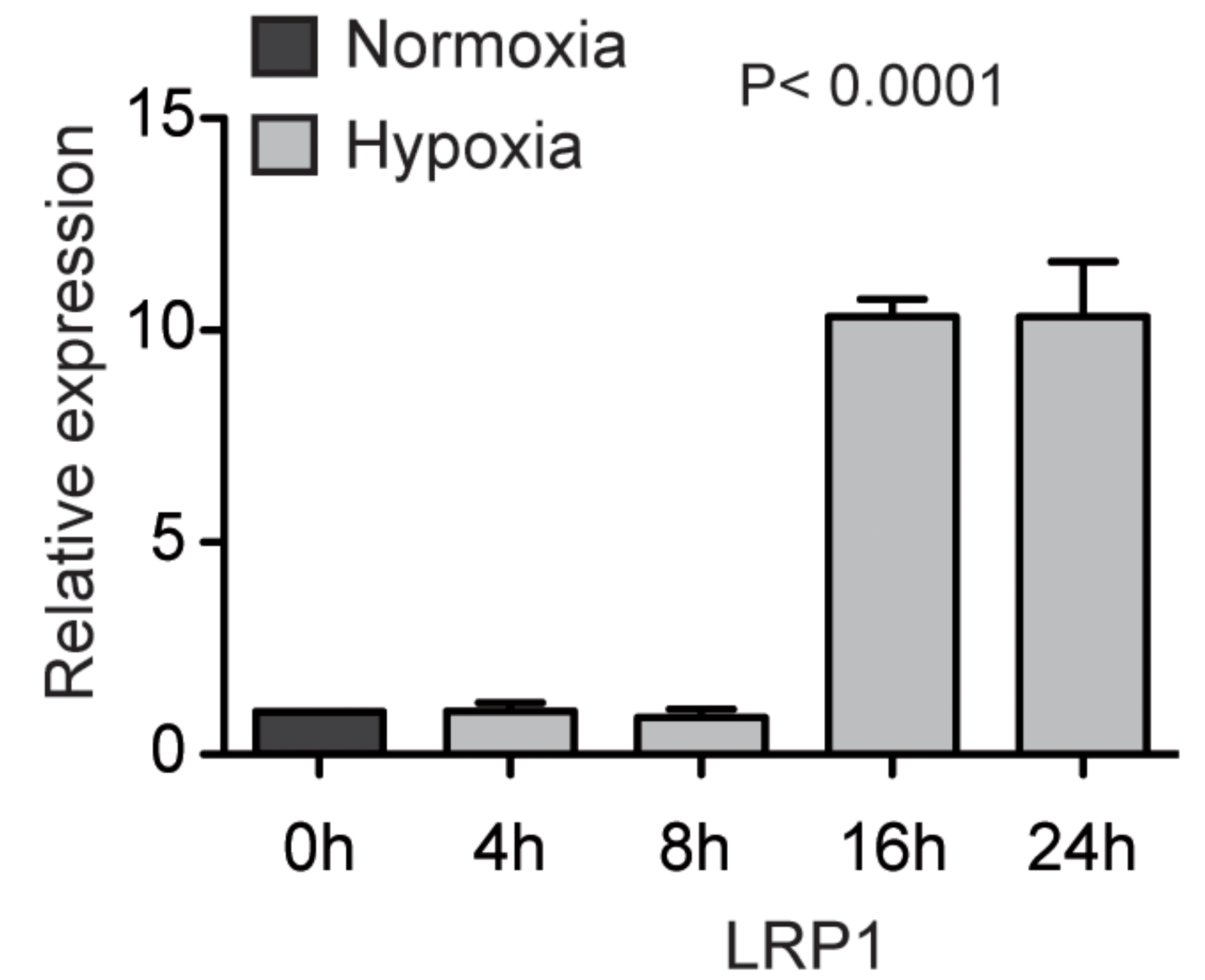

Supplement: Additional file 5: — Functional relationship between the APL subnetwork and hypoxia. Real-time PCR analysis of MMP2, KRT18, IGFBP2, ITGB2, HMOX1, and LRP1 in NB4 cells cultured in hypoxic conditions for different time points. Data represent mean values (±standard error of the mean) of three technical replicates. Significance is shown at 16 h compared with normoxia (0 h). N.s., not significant. [file 13073_2014_84_MOESM5_ESM.pdf]

### MMP2

p=7.18E-09

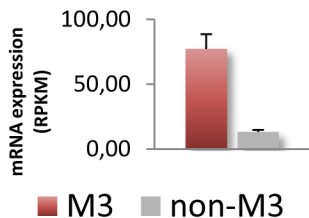

### KRT18

p=4.03E-18

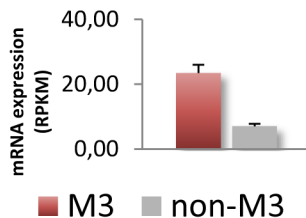

### IGFBP2

p=2.50E-25

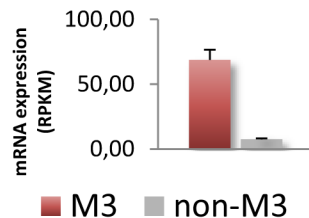

### ITGB2

p=4.94E-07

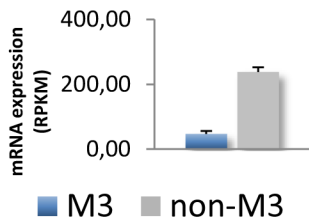

### LRP1

p=2.37E-04

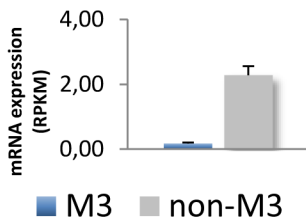

### HMOX1

p=1.00E-06

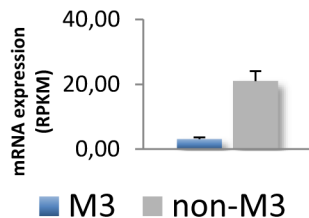

### TWIST1

p=1.33E-06

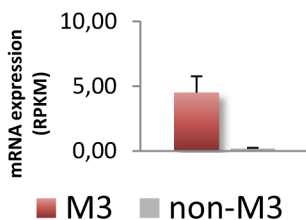

Supplement: Additional file 6: — Confirmation of APL subnetwork dysregulation using RNA-Seq data. The comparison between M3 samples (n =16) and other subtypes (n =162) is reported for MMP2, KRT18, IGFBP2, ITGB2, HMOX1, LRP1 and TWIST1. Data are expressed as RPKM. [file 13073_2014_84_MOESM6_ESM.pdf]

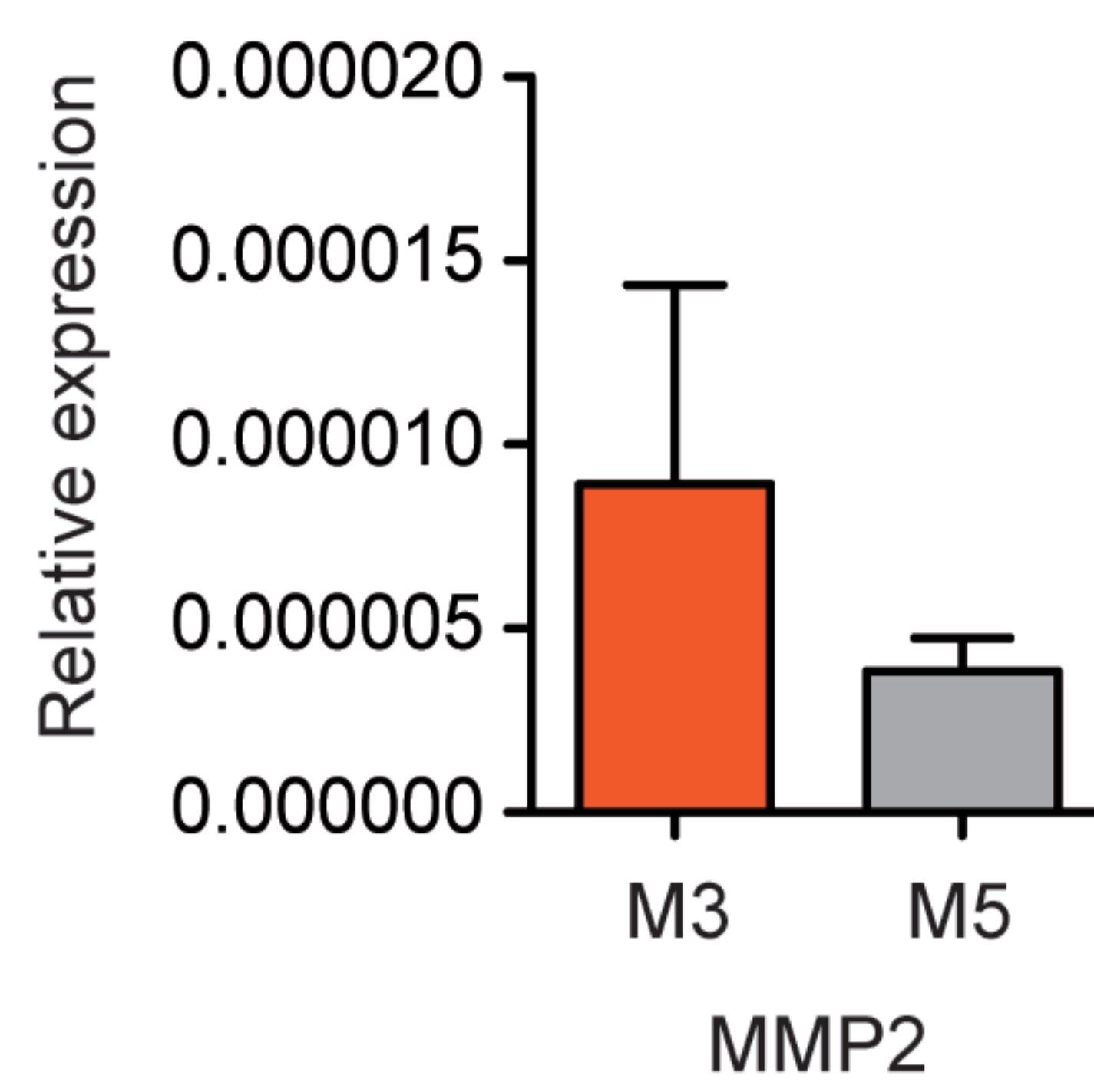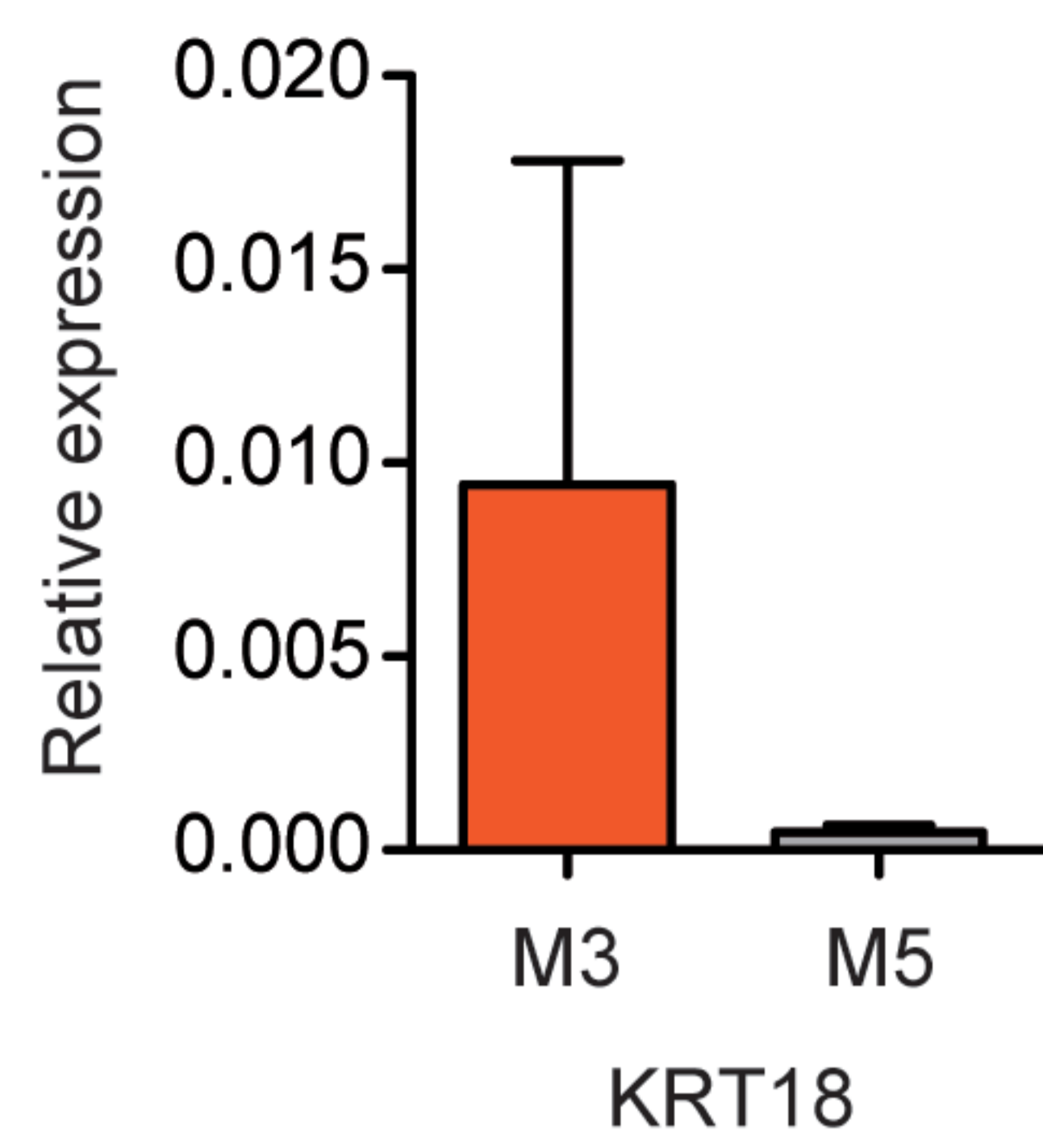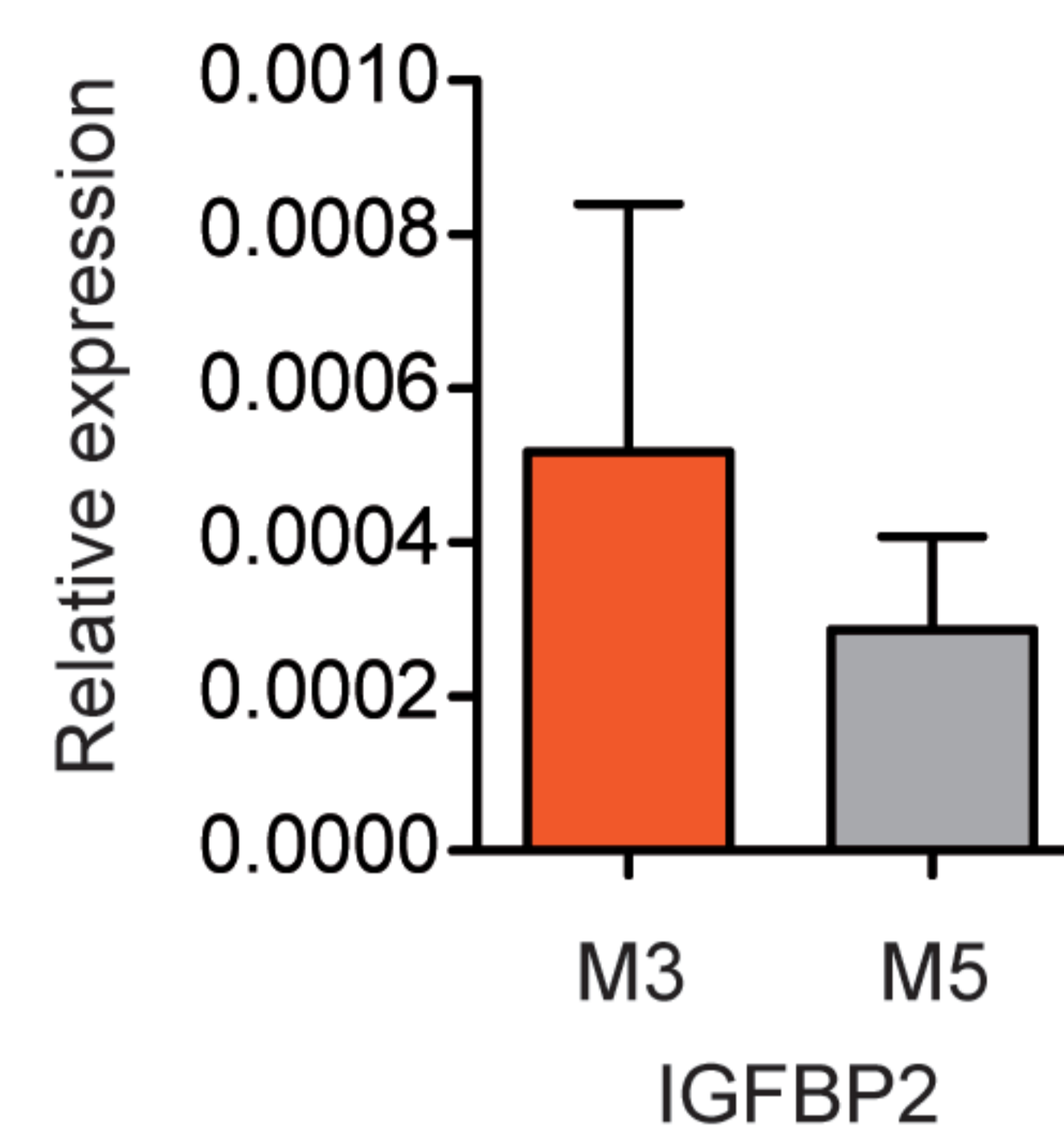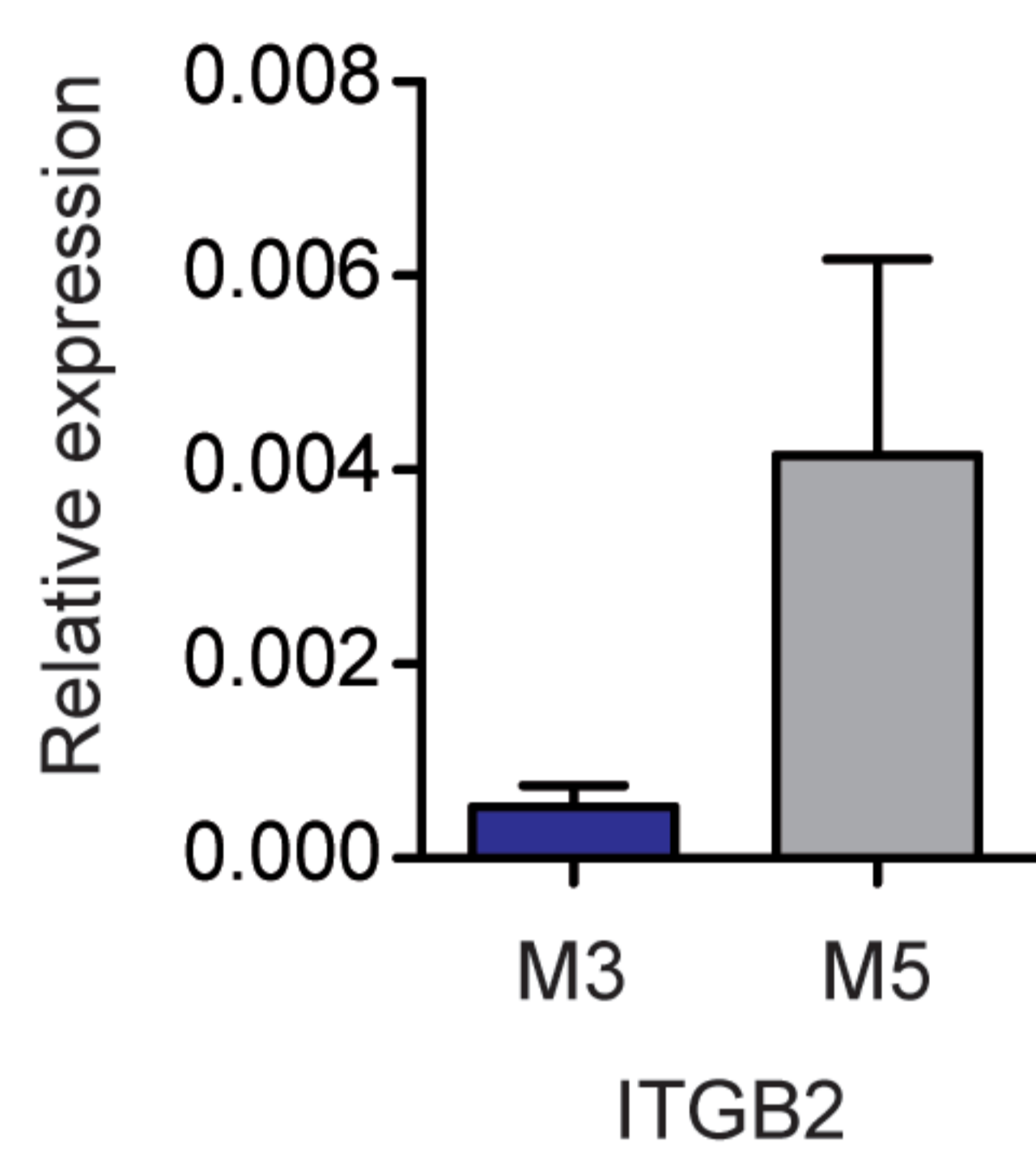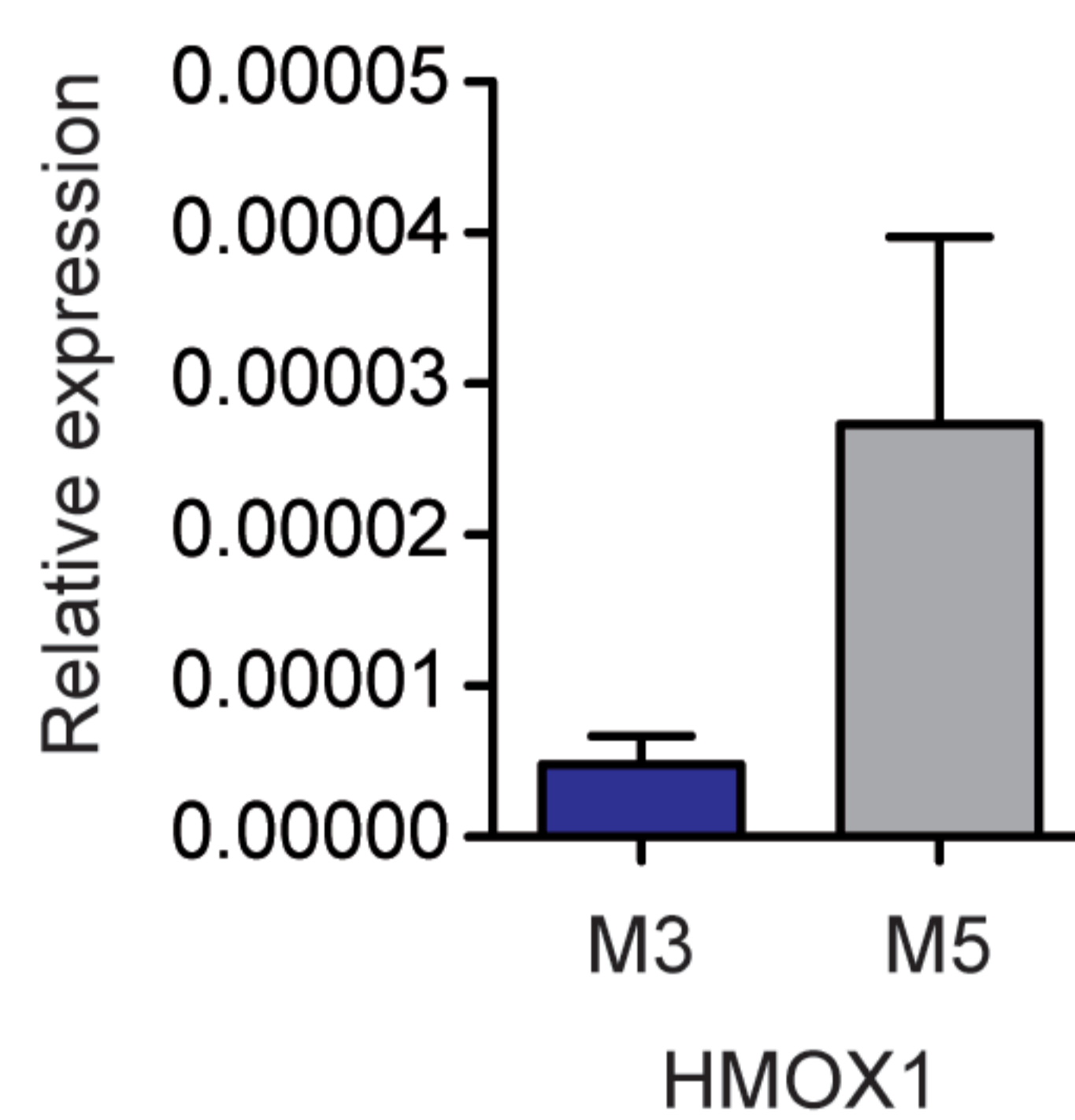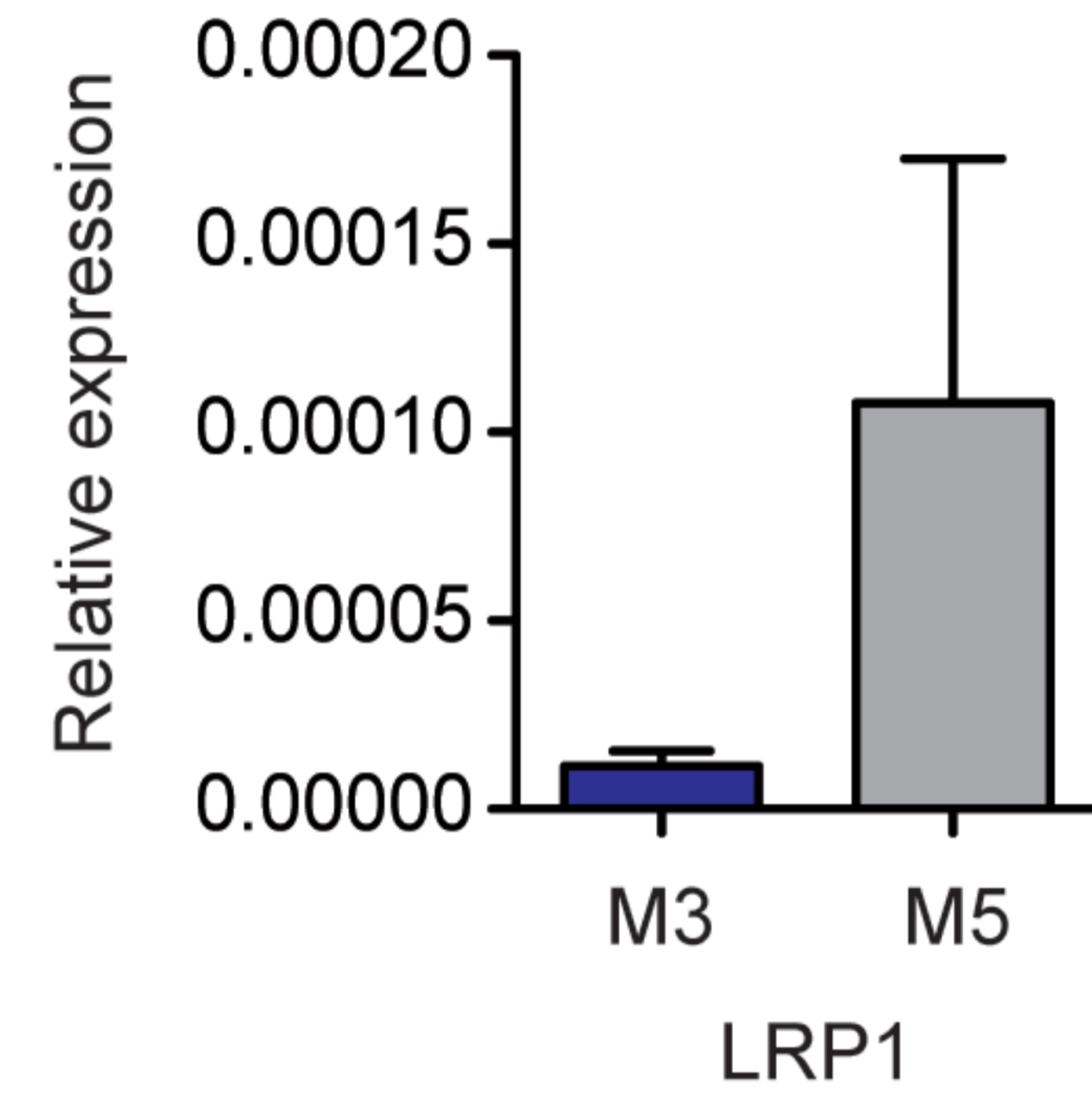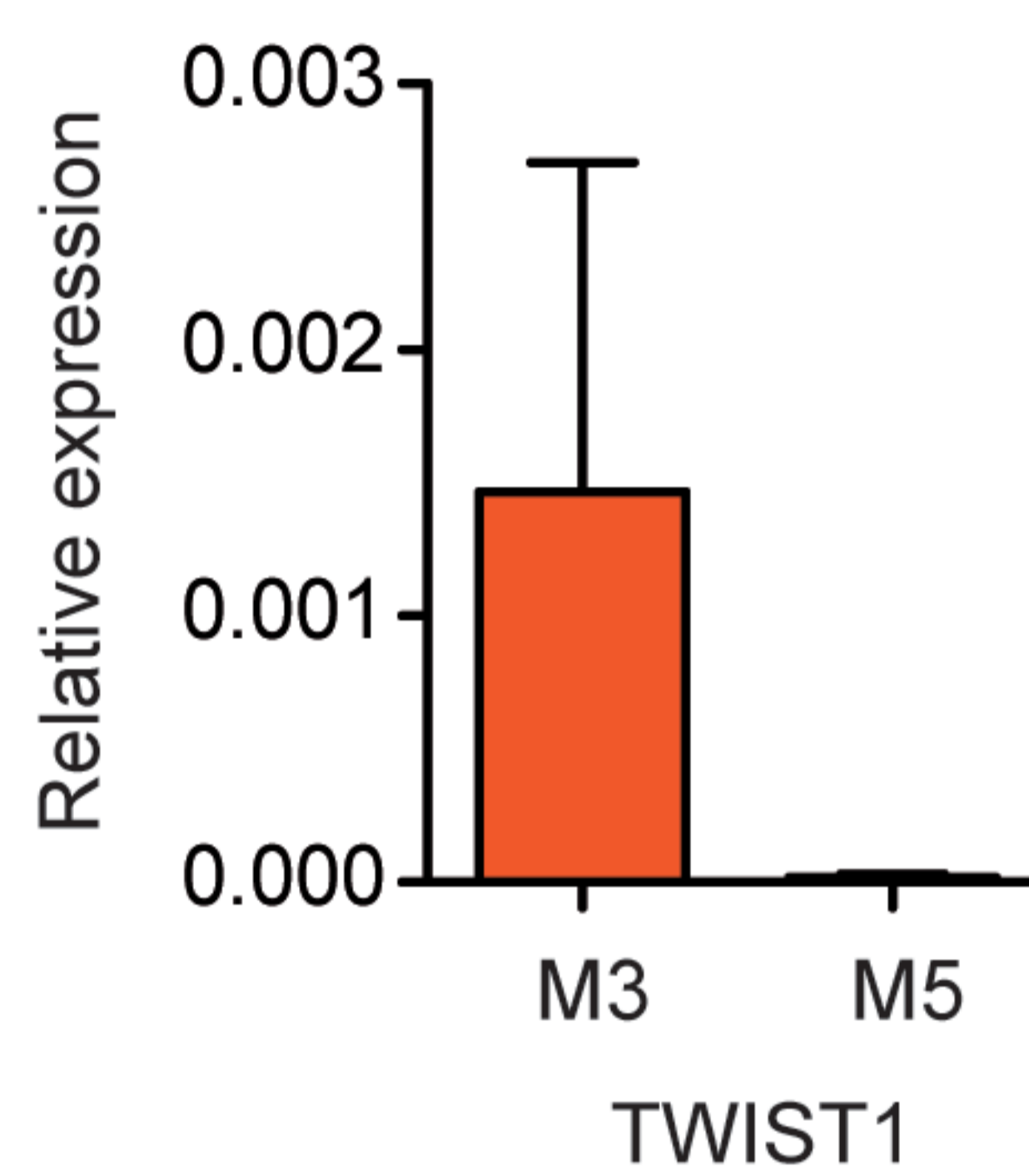

Supplement: Additional file 7: — Real-time PCR analysis of MMP2 , KRT18, IGFBP2, TGB2, HMOX1, LRP1 and TWIST1. Expression values in bone marrow samples from M3 (n =4) and M5 (n =4) patients. Differential expression is confirmed for all genes of the subnetwork and for TWIST1. [file 13073_2014_84_MOESM7_ESM.pdf]
